# Systematic review and meta-analysis on the effect of adjuvant gonadotropin-releasing hormone agonist (GnRH-a) on pregnancy outcomes in women with endometriosis following conservative surgery

**DOI:** 10.1186/s12884-024-06430-1

**Published:** 2024-04-04

**Authors:** Xuemei Qing, Lele He, Ying Ma, Yong Zhang, Wenxin Zheng

**Affiliations:** 1 Department of Obstetrics and Gynecology, Southwest Medical University, Luzhou, Sichuan 646000 China; 2 Department of Obstetrics and Gynecology, Qingbaijiang District People’s Hospital, Chengdu, Sichuan 610300 China; 3 Department of Obstetrics and Gynecology, Mianyang Central Hospital, Mianyang, Sichuan 621000 China; 4 Department of Obstetrics and Gynecology, Chongzhou Maternal and Child Health Care Hospital, Chengdu, Sichuan 611200 China; 5 Department of Obstetrics and Gynecology, Chengdu Medical College, Chengdu, Sichuan 610500 China; 6 Department of Obstetrics and Gynecology, Department of Pathology, Harold C Simmons Comprehensive Cancer Center, University of Texas Southwestern Medical Center, Dallas, TX 75390 USA

**Keywords:** Endometriosis, Conservative surgery, GnRH-a, Pregnancy rate

## Abstract

**Background:**

Endometriosis frequently results in pain and infertility. While conservative surgery offers some relief, it often falls short of ensuring satisfactory pregnancy outcomes. Adjuvant GnRH-a is administered post-surgery to mitigate recurrence; however, its impact on pregnancy outcomes remains debated. This study endeavors to assess the efficacy of adjuvant GnRH-a in enhancing pregnancy outcomes post-conservative surgery in endometriosis patients.

**Methods:**

Databases including PubMed, Embase, the Cochrane Library, Medline (Ovid), Web of Science, and Scopus were rigorously searched up to 02 August 2023, without linguistic constraints. Identified articles were screened using strict inclusion and exclusion criteria. Evaluated outcomes encompassed pregnancy rate, live birth rate, miscarriage rate, ectopic pregnancy rate, multiple pregnancy rate, mean postoperative pregnancy interval, recurrence rate, and adverse reaction rate. The Cochrane risk of bias tool and the Jadad score evaluated the included studies’ quality. Subgroup and sensitivity analysis were implemented to analyze the pooled results. A meta-analysis model expressed results as standardized mean difference (SMD) and Risk ratio (RR).

**Results:**

A total of 17 studies about 2485 patients were assimilated. Meta-analysis revealed that post-surgery, the GnRH-a cohort experienced a marginally elevated pregnancy rate (RR = 1.20, 95% CI = 1.02–1.41; *P* = 0.03) and a reduced mean time to conceive (RR = -1.17, 95% CI = -1.70- -0.64; *P* < 0.0001). Contrarily, other evaluated outcomes did not exhibit notable statistical differences.

**Conclusions:**

Incorporating adjuvant GnRH-a following conservative surgery may be deemed beneficial for women with endometriosis, especially before Assisted Reproductive Technology (ART). Nonetheless, owing to pronounced heterogeneity, subsequent research is warranted to substantiate these potential advantages conclusively.

**Registration number:**

CRD42023448280.

**Supplementary Information:**

The online version contains supplementary material available at 10.1186/s12884-024-06430-1.

## Background

Endometriosis is a chronic, inflammatory, and estrogen-dependent condition characterized by stroma and/or endometrial-like epithelium outside the endometrium and myometrium [1–3]. It manifests predominantly as persistent pelvic pain, dysmenorrhea, sexual difficulties, and notably, infertility [4, 5]. The etiology of endometriosis remains elusive with several theories proposed including retrograde menstruation, coelomic metaplasia, lymphatic and vascular metastases, and stem cell theory, among others [6–10]. Estimating the precise prevalence of endometriosis is challenging due to its varied presentations; however, it’s estimated to affect approximately 10% of reproductive-aged women globally, roughly 190 million women [11]. This prevalence can surge to 33% among those with Chronic Pelvic Pain and up to 50% in those facing infertility [12]. Such high prevalence underscores its significant toll on the quality of life and its strain on healthcare resources [13, 14].

Given the heterogeneity of endometriosis [15], its diagnosis primarily relies on surgical visualization [16], and pregnancy outcomes are also complicated. A cohort study based on the Nurses’ Health Study II investigates the association between laparoscopically confirmed endometriosis and adverse pregnancy outcomes and shows a positive association, particularly with ectopic pregnancy, spontaneous abortion, hypertensive disorders of pregnancy, and gestational diabetes mellitus [17]. Researchers have proposed a multitude of mechanistic hypotheses, such as endometrial resistance to progesterone affects oocyte quality and alters the uterine environment, resulting in poor embryo development and implantation; placental insufficiency and inadequate uterine contractility affect embryo implantation and fetal growth; and the inflammatory hypothesis is widely accepted [18]. Inflammatory and immune dysregulation mechanisms have been shown to impair endometrial tolerance and embryonic competence at the site of implantation [19]. The chronic inflammatory response of the ectopic endometrium exacerbates cell-mediated and humoral immune dysfunction in women with endometriosis, with higher levels of proinflammatory cytokines such as interleukin-1, -6, -8 and  -10, tumor necrosis factor-α, and vascular endothelial growth factor, in the peritoneal fluid of infertile women with endometriosis. All of these cytokines decrease oocyte quality, alter embryo development, and impair embryo implantation. A prospective cohort study [20], comparing uterine artery Doppler pulse indices at the time of pregnancy in patients with and without moderate-to-severe endometriosis, demonstrates the association between III-IV endometriosis and clinically measurable impaired late placental perfusion, and recommends further studies to be conducted to predict and prevent adverse pregnancy and perinatal outcomes caused by impaired late placental perfusion.

Current therapeutic approaches pivot on surgical excision of lesions and hormonal medications that suppress ovarian hormone production [21]. Hormonal medications can effectively alleviate pain symptoms [22], but their efficacy in treating infertility remains limited, often necessitating surgical interventions. Nevertheless, surgery alone doesn’t always rectify fertility issues, especially in cases of moderate to severe endometriosis [23]. Thus, a combined approach of surgical and pharmacological interventions is frequently advocated [24]. Given the plethora of available adjuvant medications, clinicians must consider the stage of endometriosis, fertility goals, other infertility determinants, and when pertinent, resort to Assisted Reproductive Technology (ART) [25, 26].

Two prominent medications in this domain are dienogest (DNG) and Gonadotropin-releasing hormone agonists (GnRH-a). Dienogest, a fourth-generation selective progestin, is typically the first line of treatment, primarily for pain relief, recurrence prevention, and contraception [27, 28]. However, its long-term use can be financially burdensome and often presents side effects like Abnormal Uterine Bleeding, which can impact quality of life and delay conception post-surgery [29]. GnRH-a, currently recognized as the most effective medicine used to treat endometriosis [30], although categorized as a second-line treatment by overseas guidelines, is commonly prescribed for short durations (3–6 months) post-surgery. In cases of low-estrogen symptoms, adjunct hormonal therapy might be necessary [31–33].

While numerous studies affirm its efficacy in pain alleviation and recurrence prevention, its impact on post-surgical pregnancy outcomes, when used as an adjuvant, remains contentious [34, 35]. Some studies extol its positive influence on clinical pregnancy rates [36], while others contradict this finding [37–39]. Amidst these conflicting results, this meta-analysis seeks to elucidate the effect of adjuvant GnRH-a on pregnancy outcomes post-conservative surgery in women with endometriosis.

## Methods

Our systematic review and meta-analysis were conducted in adherence to the Preferred Reporting Items for Systematic Reviews and Meta-Analyses (PRISMA) guidelines [40]. We registered the protocol for our study on the PROSPERO website in July 2023 under the registration number CRD 42,023,448,280.

### Search strategy

Authors Xuemei Qing and Lele He independently scoured the following electronic databases: PubMed, Embase, the Cochrane Library, Medline (Ovid), Web of Science, and Scopus, with the search spanning until 02 August 2023 and encompassing articles of all languages. Our search strategy employed MeSH terms: “endometriosis”, “Surgical Procedures, Operative”, “GnRH-a”, and “pregnancy rate”. These terms were interconnected using the logical operator “AND”, while each term was also combined with its respective synonyms using the operator “OR”. Beyond this, we scrutinized the full texts and references of pertinent reviews. Any discrepancies in our findings were deliberated upon and resolved in consultation with a third author, Yong Zhang. Given that our study neither recruited patients nor gathered personal data, ethical clearance was not mandated. The search queries show more details in Additional file 1.

### Study selection

Here we summarized the criteria for inclusion and exclusion for the study.

#### Inclusion criteria

1. Patients had confirmed endometriosis through surgery and were treated with conservative surgery.
2. The study evaluated the use of adjuvant GnRH-a post conservative surgery in women with endometriosis.
3. Outcomes were reported comparing GnRH-a usage with no GnRH-a or with alternative medications.
4. The average age of the patients in the study was under 35.
5. Studies could include conceptions achieved spontaneously or through assisted reproductive technology (ART).
6. For spontaneous conceptions, the following conditions had to be met:
  a. Anti-Müllerian hormone was at least 1.1 ng/ml.
  b. Follicle-stimulating hormone was below 12 MIU/ml.
  c. Antral follicle count on both sides was 5 or more.
7. For ART conceptions, uterine and fallopian tube factors, as well as male factors, were excluded.
8. The study design was either a randomized controlled trial (RCT) or a randomized clinical trial.
9. Full text of the article was accessible.

#### Exclusion criteria

1. Intervention trials without appropriate comparisons.
2. Studies lacking baseline comparability or where primary outcomes were not presented.
3. Non-English publications.
4. Non-original research articles.
5. Duplications of previously published studies.

For studies that were repeated, we opted for the publication with the most extensive sample size or the most recent publication date.

Initially, search results were scanned for duplicate publications using titles and abstracts. We then refined our selection by assessing articles according to the inclusion and exclusion criteria. We excluded articles that were reviews, meta-analyses, guidelines, conference summaries, letters to the editor, animal studies, case reports or series, observational studies, non-RCTs, and those deemed irrelevant. Post initial screening, full texts were carefully reviewed for comprehensive qualitative and quantitative analysis. Specific reasons for exclusions after full-text review can be found in Additional file 2.

### Quality assessment

All studies included in our review were either randomized controlled trials (RCTs) or randomized clinical trials. Authors Xuemei Qing and Lele He independently assessed the quality of these studies using both the Cochrane Risk of Bias Assessment Tool and the Jadad score.

The Cochrane Risk of Bias tool evaluates several aspects:

1. Random sequence generation
2. Allocation concealment
3. Blinding of participants and personnel
4. Blinding of outcome data
5. Completeness of outcome data
6. Selective reporting
7. Potential other biases

For each aspect, the risk is categorized as either low, unclear, or high. A study was deemed to be of high quality if there was no high risk of bias in any category. Fair quality was assigned to studies with one high risk or two unclear risks. Any study not meeting these criteria was considered to be of poor quality.

The Jadad score assesses studies based on:

1. Randomization
2. Blinding
3. Loss of follow-up
4. Withdrawal

A score between 1 and 3 denotes low quality, while a score between 4 and 7 indicates high quality.

All quality evaluations are listed in Additional file 3.

### Data extraction

Authors Xuemei Qing and Lele He were responsible for independently extracting pertinent information from each included study. In cases where disagreements arose, the points of contention were discussed, and if a consensus couldn’t be reached, the third author, Yong Zhang, was consulted.

The extracted data encompassed as follows:

1. First author’s name
2. Year of publication
3. Country where the study was conducted
4. Study design
5. Disease type
6. Drug regimens used in both the GnRH-a and control groups
7. Sample size
8. Duration of therapy
9. Follow-up period
10. Outcomes reported
11. Method of conception

Regarding the outcome indicators:

1. The primary outcome focused on the pregnancy rate.
2. Secondary outcomes delved into live birth rate, miscarriage rate, ectopic pregnancy rate, multiple pregnancy rate, and the mean postoperative pregnancy interval.
3. Additional outcomes covered rates of recurrence and adverse reactions.

The essential characteristics of included studies are cataloged in Additional file 4.

### Statistical analysis

We conducted all data analyses using RevMan 5.4 software. This meta-analysis evaluated multiple outcomes: For dichotomous outcomes, we calculated the Risk Ratio (RR) along with its 95% confidence intervals (CI); For continuous outcomes, the standardized mean difference (SMD) with 95% CI was computed. When the original data reported the median with range or interquartile range, we estimated the mean and standard deviation.

Statistical significance was set at *P* < 0.05. Heterogeneity across studies was assessed using the Q-test and I^2^ index: I^2^ of 0–25% indicated low heterogeneity; I^2^ of 25–50% denoted moderate heterogeneity; I^2^ of 50–75% represented substantial heterogeneity; and I^2^ above 75% signified high heterogeneity.

A random-effects model was employed when the Q-test resulted in *P* < 0.1 or I^2^ value ≥ 50%. Otherwise, a fixed-effect model was utilized. Subgroup analyses were carried out based on variations in GnRH-a regimens, different control groups, the involvement of assisted reproductive technology (ART) and study quality in pregnancies. Notably, pooled analysis for subgroups was conducted only when at least two studies existed within a subgroup. Detailed subgroup statistical analyses are available in Additional file 5.

To evaluate the robustness of our results, we conducted a sensitivity analysis by omitting one study at a time. Publication bias was inspected using funnel plots.

## Results

### Selection of studies

**Fig. 1:**
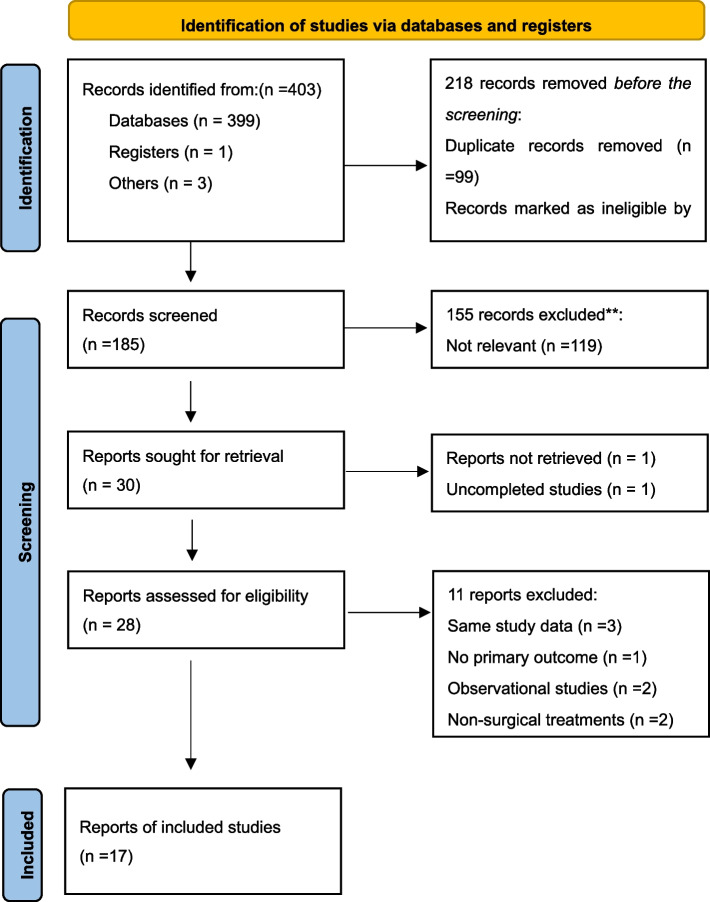
Flowchart of the study selection

A total of 403 articles were identified, duplicates were excluded (*n* = 99), then records marked as ineligible by automation tools were excluded (*n* = 119), which included review and meta-analysis (*n* = 96), conference or meeting (*n* = 8), animal experiment (*n* = 1), case report or series (*n* = 3), letters to the editor (*n* = 5), guideline (*n* = 2), and non-English publication (*n* = 4). The remaining 185 studies were further evaluated by reading the titles and abstracts, excluding irrelevant studies (*n* = 119) and other reasons (*n* = 36). Efforts were made to obtain the full text of the remaining 30 documents for re-screening. 1 article of full text could not be obtained, and 1 study is not completed which is recruiting. we read further the 28 full texts, and 11 of them were excluded again, due to the same study data (*n* = 3), no primary outcome (*n* = 1), observational studies (*n* = 2), non-surgical treatments (*n* = 2), and control measures are inconsistent (*n* = 3). Finally, 17 studies were included for meta-analysis. The flowchart of the study selection is shown in Fig. 1.

### Characteristics of the included studies and quality assessment

All of the studies are published in English. The publication year ranges from 1999 to 2022. Of the seventeen included trials, 1 was done in America [41], 8 in Europe [38, 42–48], 8 in Asian [36, 37, 49–54]. Seventeen studies included two GnRH-a proposal groups: five long GnRH-a proposals [43, 47, 48, 53, 54] and twelve short proposals [36–38, 41, 42, 44–46, 49–52]. Twelve studies used a blank control [36–38, 41, 42, 44–46, 49–52], and five studies used other drugs [43, 47, 48, 53, 54]. It should be clarified that the control group varied and two studies included three comparison groups [37, 52], so we divided them into two comparison groups and analyzed them separately. Thus, we obtained 19 data sets for this analysis. Seven studies with ART [41, 44, 45, 47, 49–51],three studies by spontaneous pregnancy [36, 43, 46], and one of them [43] included conception both by spontaneously and ART. Because the proportion of ART pregnancies was very small, we classified it in the spontaneously group. Only one study [37] reported pregnancy outcomes by stage of endometriosis, so subgroup analysis of stage couldn’t be conducted.

With a total of 2485 patients, 1200 cases in the GnRH-a group used different GnRH-a products: Triptorelin in 6 studies [37, 41–45], Leuprorelin in 2 studies [41, 46], Leuprodex in 1 studies [47], Leuprolide in 2 studies [38, 48], Goserelin in 3 studies [36, 49, 50], the remaining studies didn’t indicate the drug name; different injection methods (IM. or IH.) and medication dose (3.75 mg or 3.6 mg); but there was no significant difference on therapeutic effect. And 1285 cases in the control group included the blank and other medications control (Letrozole [37], Dienogest [41], Medroxyprogesterone [51], GnRH-ant [52], Gestrinone/Mifepristone [44], and Chinese herbal medicine(CM) [53]). The GnRH-a and the control group met baseline comparability in the patient characteristics. Most included studies were followed for more than one year, some longer than two years, and some studies were followed within six months. Due to different methods to report the age, infertility duration and type (i.e., mean with SD; median with range), we did’ t analysis the results across the studies in this meta-analysis. The characteristics of the included studies are listed in Table 1.

**Table 1:** Characteristics of the included studies in the meta-analysis

Study	Country	Jadad score	GnRH-a group	Control group	Sample size	Time of Therapy	Follow-up	Outcome^a^	conception way
Saeed Alborzi 2010 [37]	Iran	5	Triptorelin 3.75 mg	Without GnRH-a/ Letrozole	40/57 /47	2 M	12 M	afg	unclear
Ibrahim Alkatout 2012 [46]	Germany	5	Leuprorelin 3.75 mg	Without GnRH-a	148/137	3 M	2Y	abcd	unclear
Piyush Bansal 2018 [47]	India	7	Leuprodex 3.75 mg	Without GnRH-a	41/42	1 M	2 Y + 6 M	ab	SO + IUI
M.Busacca 2001 [38]	Italy	5	Leuprolide 3.75 mg	Without GnRH-a	44/45	3 M	6-36 M	ah	unclear
Marcello Ceccaroni 2021 [41]	Italy	3	Triptorelin/ Leuprorelin 3.75 mg	Dienogest	81/65	6 M	30 ± 6 M	abh	ART/spontaneously
W. Decleer 2016 [54]	Belgium	6	GnRH-a	Without GnRH-a	61/58	3 M	unclear	a	ART
Haiyan Guo 2022 [51]	China	5	GnRH-a	Medroxyprogesterone	150/150	1 M	unclear	abcde	ART
Giuseppe Loverro 2006 [43]	Italy	3	Triptorelin 3.75 mg	Without GnRH-a	29/25	3 M	5Y	ag	spontaneously
Recai Pabuccu 2007 [52]	Turkey	5	GnRH-a	GnRH-ant	48/50	1 M	unclear	ac	ART
Dagmar Rickes 2002 [49]	Germany	5	Goserelin 3.6 mg	Without GnRH-a	55/55	6 M	4 w	a	ART
Elisabet Rodr´ıguez-Tarrega 2020 [42]	Italy	7	Triptorelin 3.75 mg	placebo	91/92	3 M	unclear	abcde	ART
Eric S. Surrey 2002 [48]	California	5	Leuprolide 3.75 mg	Without GnRH-a	25/26	3 M	unclear	af	ART
Paolo Vercellini 1999 [50]	Italy	5	Goserelin 3.6 mg	Without GnRH-a	107/103	6 M	2Y	ag	unclear
Huiling Xue 2018 [44]	China	4	Triptorelin 3.75 mg	Gestrinone/ Mifepristone	50/50 /50	3 M	2Y	abcdgh	unclear
Xin-hua Yang 2014 [36]	China	2	Goserelin (Zoladex) 3.6 mg	Without GnRH-a	63/62	3 M	18 M	afg	spontaneously
Yu Yang 2019 [45]	China	2	Triptorelin 3.75 mg	Without GnRH-a	65/65	6 M	2Y	agh	unclear
ZHAO Rui-hua 2012 [53]	China	2	GnRH-a 3.75 mg	CM	102/106	6 M (stage I-II:3 M)	20.66 ± 6.75/20.83 ± 6.65 M	afgh	unclear

^a^ a: pregnancy rate, b: live birth rate, c: miscarriage rate, d: ectopic pregnancy rate, e: multiple pregnancy rate, f: the mean interval from surgery to pregnancy, g: recurrence rate, h: adverse reactions rate

**Fig. 2:**
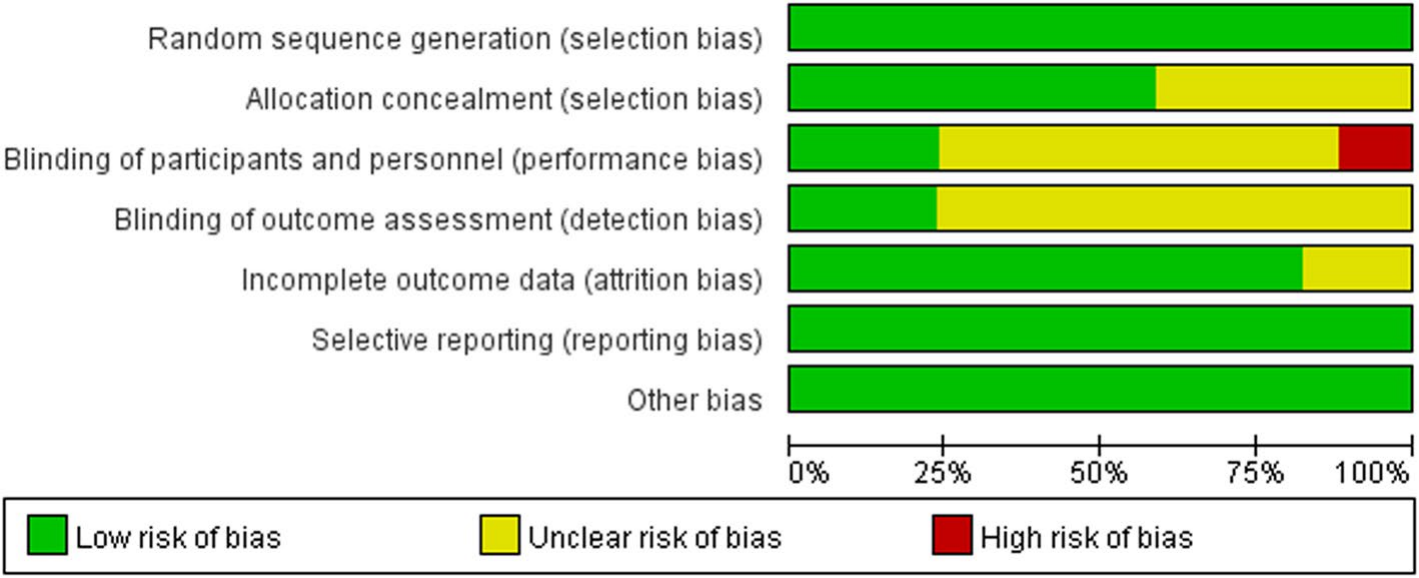
Risk of bias graph

**Fig. 3:**
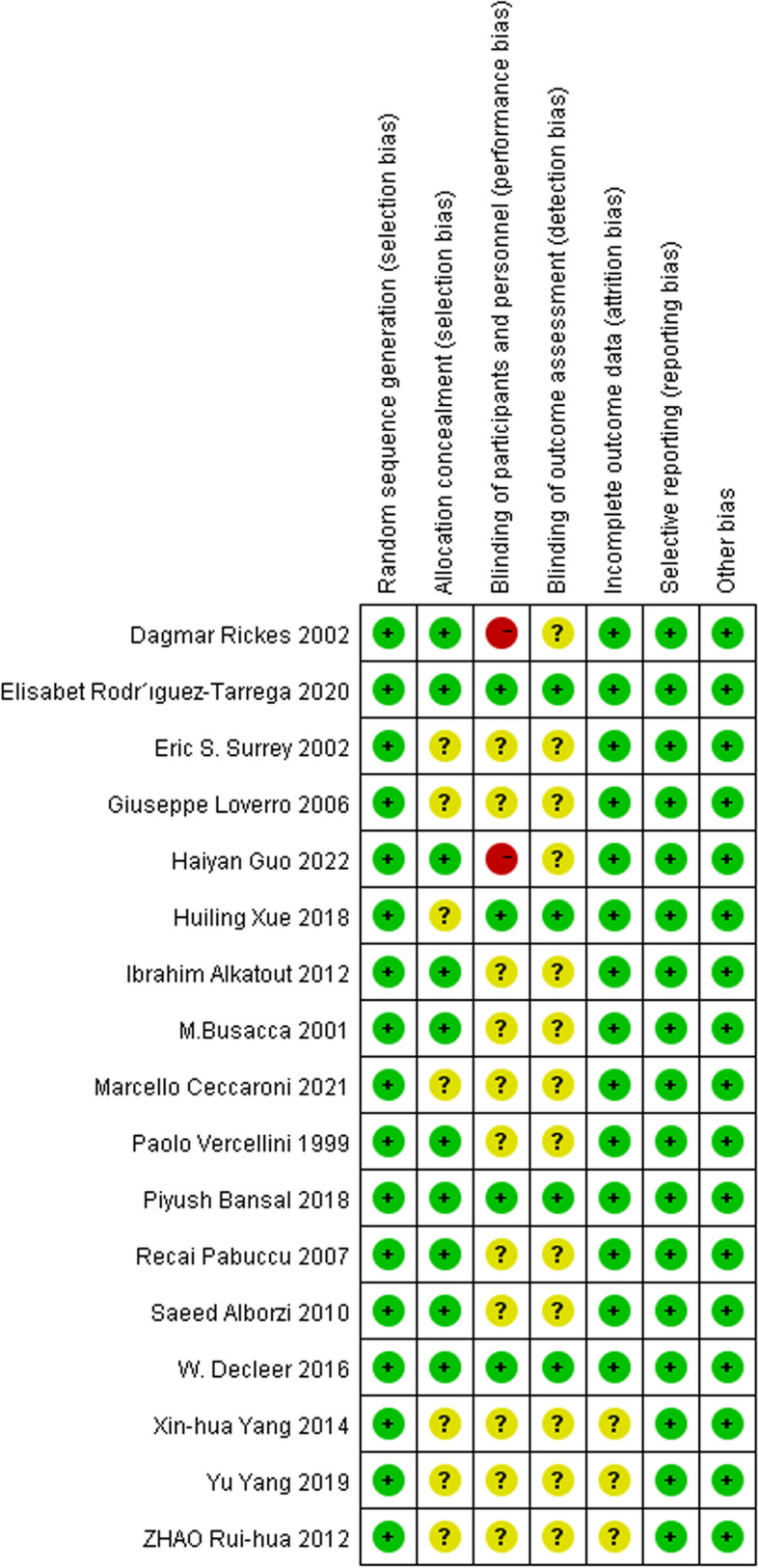
Risk of bias summary

The quality of studies ranged from low to high, two studies [42, 47] were up to score 7, three studies [36, 45, 53] were as low as score 2. The overall level of research was at a moderate level with the median points of the Jadad score 4.5. The quality of each trial was evaluated by the Cochrane risk of bias tool shown in Figs. 2 and 3.

### Primary outcome

**Fig. 4:**
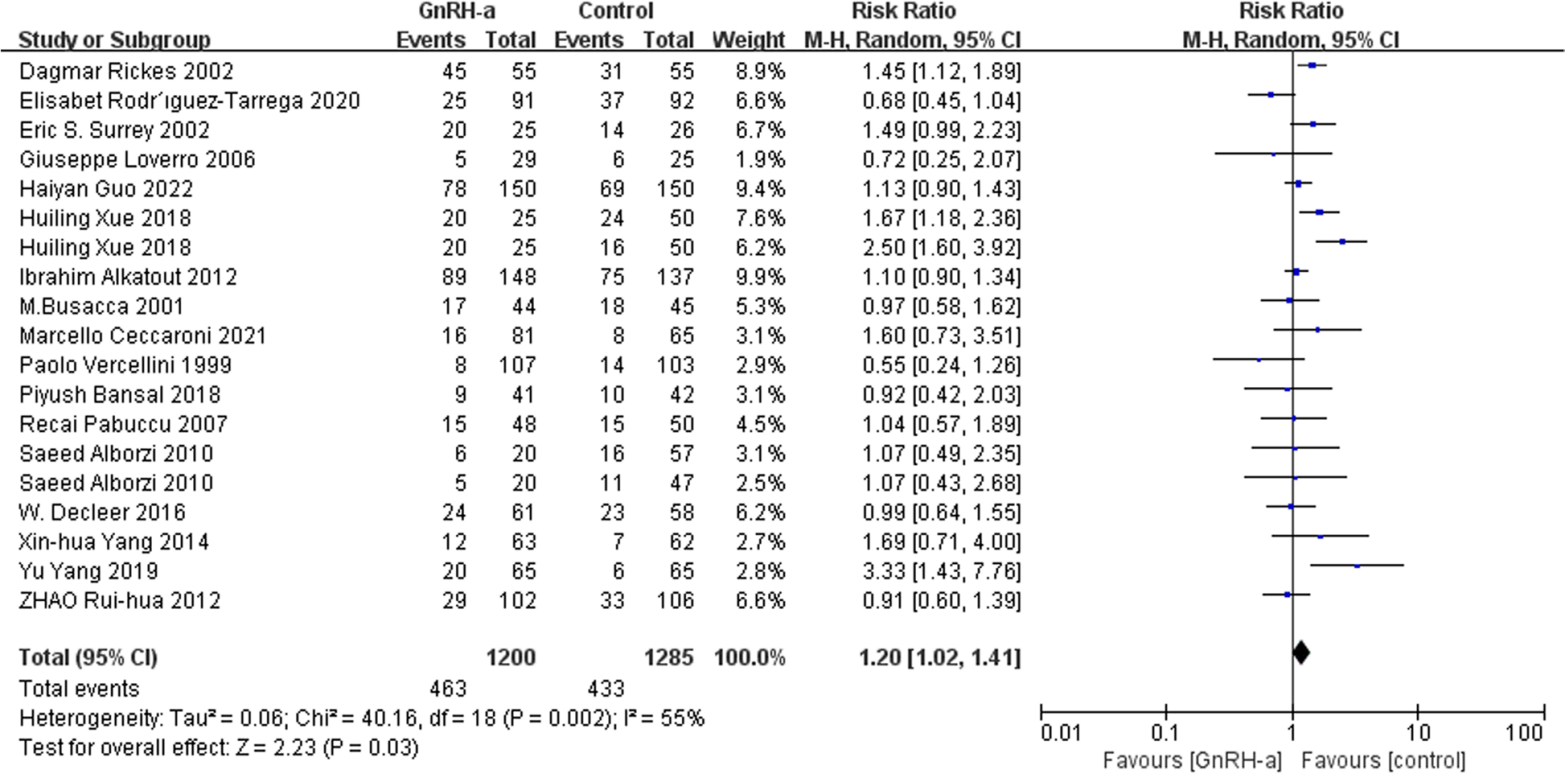
Forest plot of the effect of GnRH-a on pregnancy rate

We chose the random-effects model due to moderate heterogeneity for effects on pregnancy rate. The result showed that there was a slightly higher pregnancy rate between the GnRH-a and control groups after surgery (RR = 1.20, 95% CI = 1.02–1.41; *P* = 0.03, I^2^ = 55%) (Fig. 4).

### Secondary outcomes

**Fig. 5:**
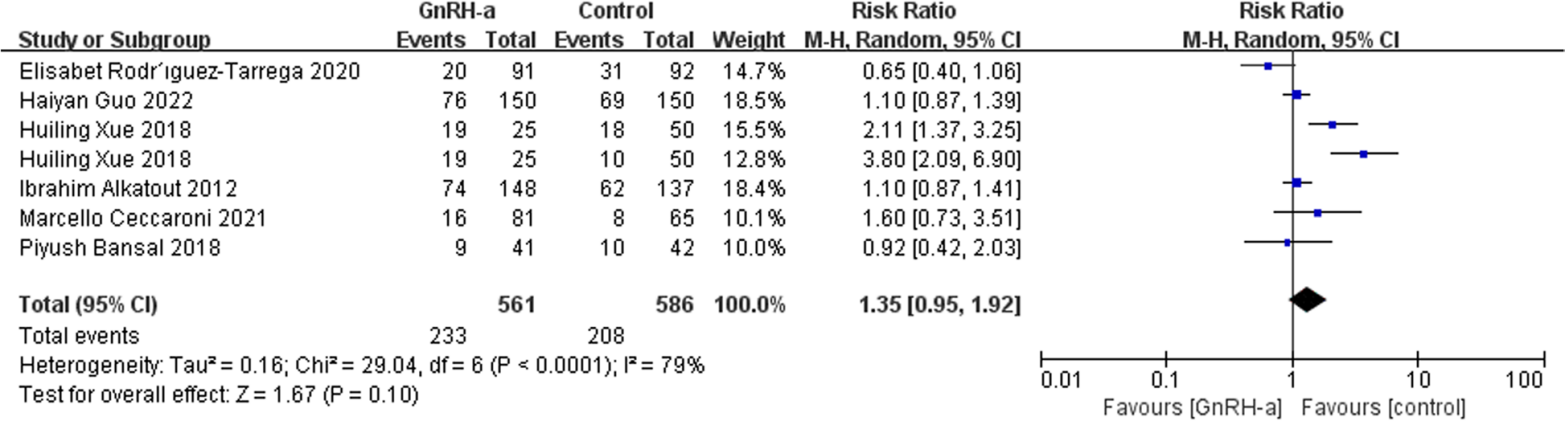
Forest plot of the effect of GnRH-a on live birth rate

**Fig. 6:**
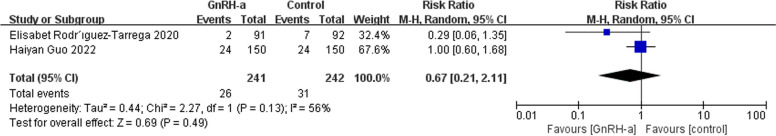
Forest plot of the effect of GnRH-a on multiple pregnancy rate

**Fig. 7:**
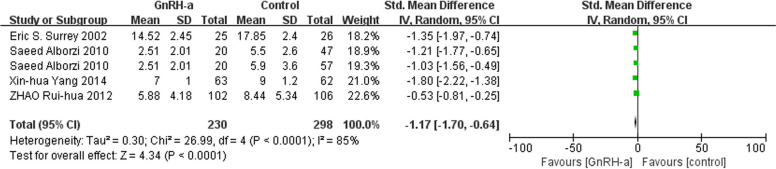
Forest plot of the effect of GnRH-a on the mean interval from surgery to pregnancy

We chose the random-effects model due to moderate to high heterogeneity for effects on live birth rate, multiple pregnancy rate and the mean interval from surgery to pregnancy. There was no difference in live birth rate (RR = 1.35, 95% CI = 0.95–1.92; *P* = 0.10, I^2^ = 79%) (Fig. 5) and multiple pregnancy rate (RR = 0.67, 95% CI = 0.21–2.11; *P* = 0.49, I^2^ = 56%) (Fig. 6). Mean postoperative pregnancy interval appeared shorter in the GnRH-a group compared with the control group (RR = -1.17, 95% CI = -1.70- -0.64; *P* < 0.0001, I^2^ = 85%) (Fig. 7).

**Fig. 8:**
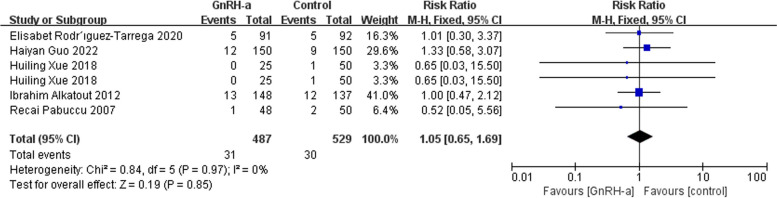
Forest plot of the effect of GnRH-a on miscarriage rate

**Fig. 9:**
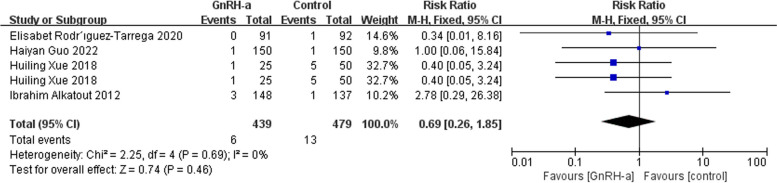
Forest plot of the effect of GnRH-a on ectopic pregnancy rate

Then we used the fixed-effects model due to low heterogeneity for effects on miscarriage rate and ectopic pregnancy rate. However, there was no significant significance in terms of miscarriage rate (RR = 1.03, 95% CI = 0.64–1.66; *P* = 0.92, I^2^ = 0%) (Fig. 8) and ectopic pregnancy rate (RR = 0.69, 95% CI = 0.26–1.85; *P* = 0.46, I^2^ = 0%) (Fig. 9).

### Supplementary outcomes

**Fig. 10:**
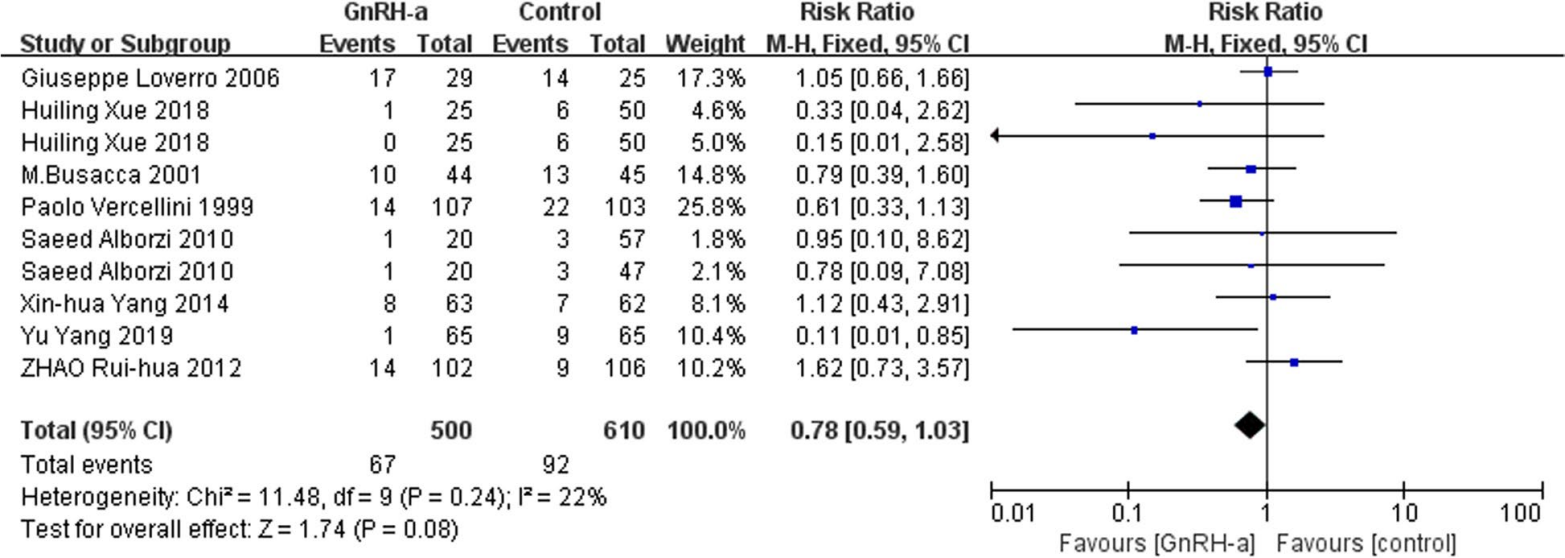
Forest plot of the effect of GnRH-a on recurrence rate

**Fig. 11:**
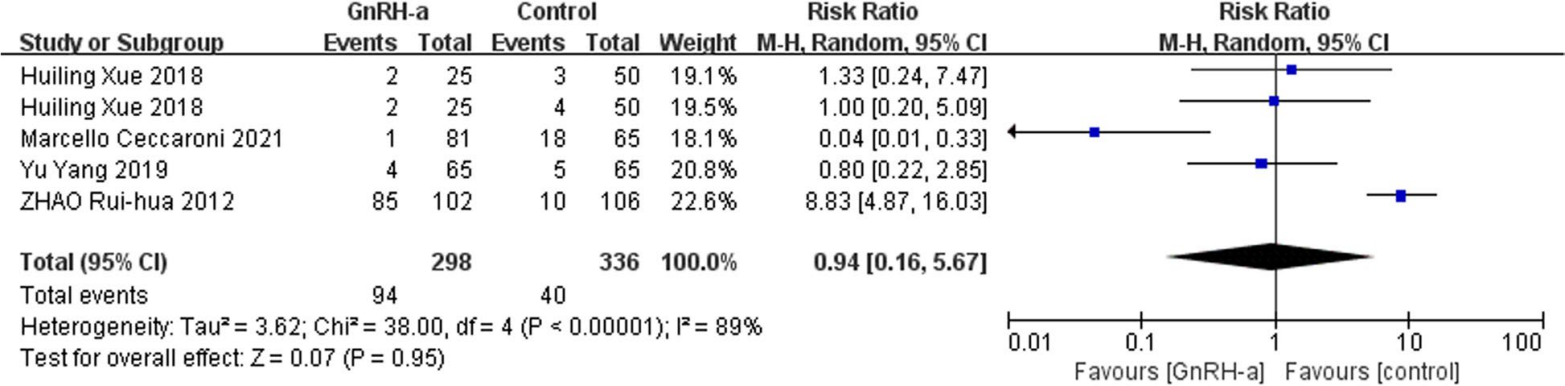
Forest plot of the effect of GnRH-a on adverse reactions rate

We used the fixed-effects model due to low heterogeneity for effects on recurrence rate and the random-effects model because of high heterogeneity for adverse reactions rate. Meta-analysis showed that there was no difference on the recurrence rate (RR = 0.78, 95% CI = 0.59–1.03; *P* = 0.08, I^2^ = 22%) (Fig. 10) and adverse reactions rate (RR = 0.94, 95% CI = 0.16–5.67; *P* = 0.95, I^2^ = 89%) (Fig. 11).

### Subgroup analysis

The included studies in this meta-analysis were heterogeneous in terms of study protocol: different GnRH-a proposals, control groups, whether with ART, and different study quality; and we conducted subgroup analysis based on these protocol differences. The results of the subgroup analysis are shown in Table 2.

**Table 2:** Subgroup analysis of pregnancy rate and live birth rate

Subgroup	No. of groups	RR (95% CI)	*P* Value	P for heterogeneity	I^2^ (%)	P for between Subgroup heterogeneity
**Pregnancy rate**					55%	
**GnRH-a group**						0.90
Long proposal	5	1.03(0.83–1.28)	0.76	0.67	0	
Short proposal	14	1.02(0.92–1.12)	0.74	1.00	0	
**Control group**						0.56
Blank	12	0.99(0.87–1.13)	0.87	0.98	0	
Other medications	7	1.05(0.93–1.18)	0.49	0.97	0	
**Whether ART**						0.62
ART	7	1.01(0.91–1.13)	0.83	0.88	0	
spontaneously	3	1.13(0.73–1.75)	0.73	0.96	0	
**Study quality**						0.88
High quality	14	1.02(0.92–1.11)	0.74	0.99	0	
Low quality	5	1.04(0.78–1.39)	0.79	0.93	0	
**live birth rate**					79%	
**GnRH-a group**						0.48
Long proposal	1	1.23(0.73–2.06)	0.44	NA	NA	
Short proposal	6	1.01(0.88–1.16)	0.87	0.76	0	
**Control group**						0.30
Blank	3	0.97(0.81–1.15)	0.71	0.54	7.6	
Other medications	3	1.12(0.90–1.39)	0.30	0.88	0	
**Whether ART**						0.38
ART	3	0.96(0.79–1.16)	0.65	0.55	0	
spontaneously	1	1.23(0.73–2.06)	0.44	NA	NA	
**Study quality**						0.48
High quality	6	1.01(0.88–1.16)	0.87	0.78	0	
Low quality	1	1.23(0.73–2.06)	0.44	NA	NA	

Using the fixed-effects model, the pooled RR of pregnancy rate in the long and short GnRH-a proposals was 1.02 (95%CI = 0.93–1.11; *P* = 0.70). By the same method, the results of other subgroup analyses were as follows: the pooled results of blank and other medications control groups were same with GnRH-a proposals. The pooled RR of conceive by ART and spontaneously was 1.02 (95%CI = 0.91–1.14; *P* = 0.73). About study quality: the pooled results were (RR = 1.02, 95%CI = 0.93–1.11; *P* = 0.70).

The fixed model produced the similar result on live birth rate. By the same method, the pooled results in the long and short GnRH-a proposals were (RR = 1.02, 95%CI = 0.90–1.17; *P* = 0.72); the pooled RR in blank and other medications control groups were same with GnRH-a proposals. Conceive by ART and spontaneously, the pooled results were (RR = 0.99, 95%CI = 0.82–1.18; *P* = 0.87). About study quality: the pooled RR was 1.02 (95%CI = 0.90–1.17; *P* = 0.72).

The analysis confirmed the source of heterogeneity, however, we couldn’t rule out them. Furthermore, only one study in some subgroups on the live birth rate which made the results of this subgroup analysis potentially unstable. We did not conduct subgroup analysis in effects of other terms, because miscarriage rate, ectopic pregnancy rate and recurrence rate are low heterogeneous, and less than two studies were in each subgroup on the effect of multiple pregnancy rate, mean interval from surgery to pregnancy and adverse reactions rate.

### Sensitivity analysis and publication bias

**Fig. 12:**
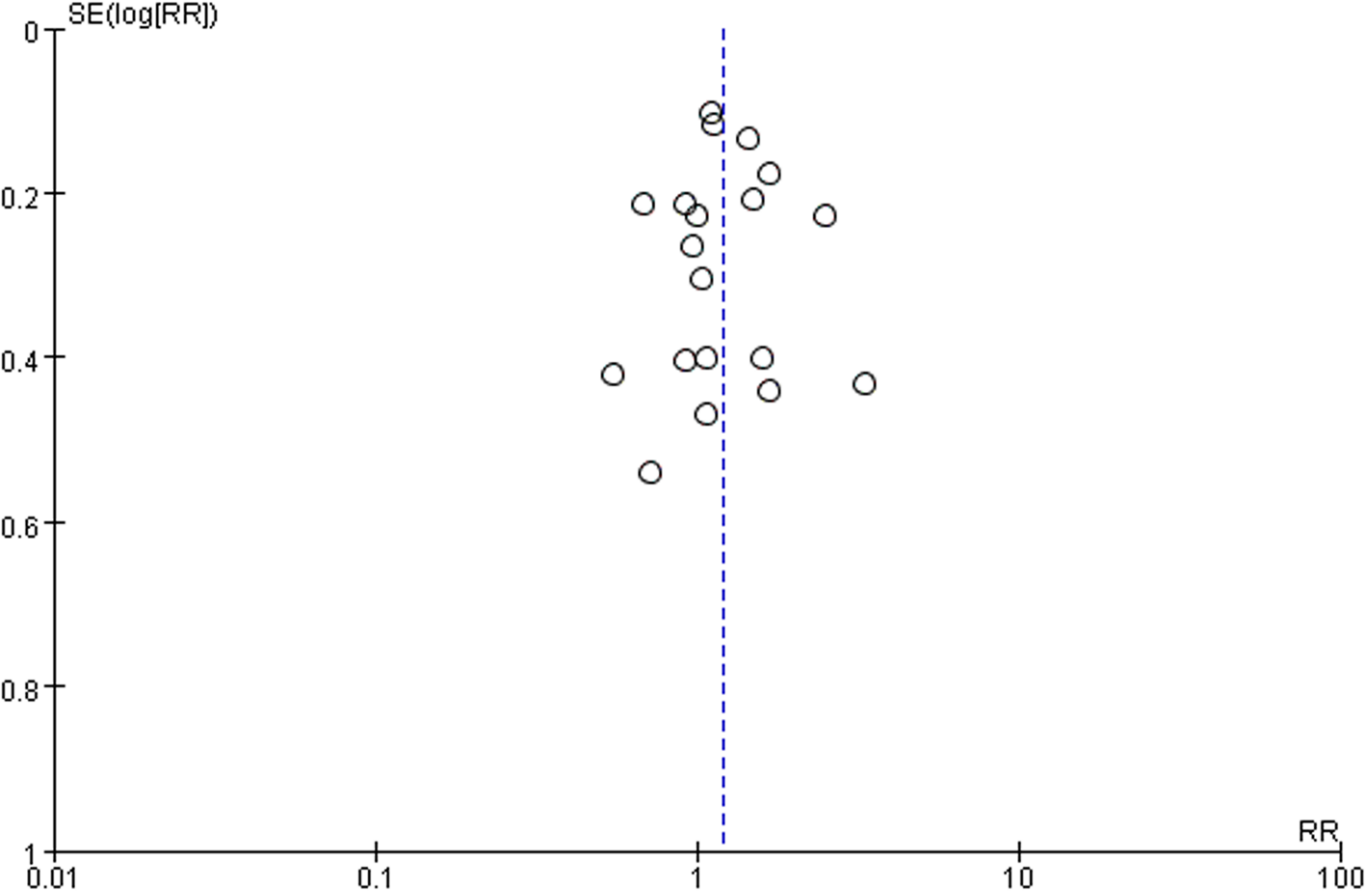
Funnel plot of the included studies in the meta-analysis of pregnancy rate

Sensitivity analyses were used to assess the stability of meta-analyses. When either study was removed, there was no change in overall statistical significance between pregnancy rate with RR from 1.022(95%CI = 1.03–1.44) to 1.25(95%CI = 1.07–1.46), indicating relatively stable results from this meta-analysis (Table 3). The results showed no significant bias in pregnancy rate (Fig. 12). Egger’s test *P* = 0.969 (> 0.05) and Begg’s test *P* = 0.834(> 0.05), both confirming insignificant bias. As fewer than ten included studies, we did not perform sensitivity analyses and publication bias assessments for other outcomes.

**Table 3:** Sensitivity analysis of pregnancy rate

Excluded study	pregnancy rate	
	RR	95%CI
None	1.20	1.02–1.41
Saeed Alborzi 2010	1.21	1.03–1.43
Saeed Alborzi 2010	1.21	1.02–1.42
Ibrahim Alkatout 2012	1.21	1.01–1.46
Piyush Bansal 2018	1.21	1.03–1.43
M.Busacca 2001	1.022	1.03–1.44
Marcello Ceccaroni 2021	1.19	1.01–1.41
W. Decleer 2016	1.22	1.03–1.44
Haiyan Guo 2022	1.21	1.01–1.45
Giuseppe Loverro 2006	1.21	1.03–1.43
Recai Pabuccu 2007	1.21	1.02–1.43
Dagmar Rickes 2002	1.18	0.99–1.41
Elisabet Rodr´ıguez-Tarrega 2020	1.25	1.07–1.46
Eric S. Surrey 2002	1.18	1.00-1.40
Paolo Vercellini 1999	1.23	1.05–1.44
Huiling Xue 2018	1.17	0.99–1.38
Huiling Xue 2018	1.15	0.99–1.33
Xin-hua Yang 2014	1.19	1.01–1.41
Yu Yang 2019	1.17	1.00-1.37
ZHAO Rui-hua 2012	1.23	1.04–1.45

## Discussion

Our meta-analysis results indicate that the use of adjuvant GnRH-a following conservative surgery in women with endometriosis appears to enhance the pregnancy rate, especially within long GnRH-a protocols. However, the outcomes related to live birth rates were not statistically significant [12]. The established relationship between endometriosis and infertility points towards several factors, including inflammation, immune disturbances, decreased ovarian reserve, surgical complications, and a significant recurrence rate [55]. While surgical interventions might offer benefits in terms of pregnancy [56, 57], they often fail to secure optimal outcomes. Skilled surgeons are crucial, as inexperienced hands can negatively influence IVF cycle cancellation rates [58]. Disturbingly, recurrence rates post-surgery can reach up to 50% within five years [26].

Gonadotropin-releasing hormone (GnRH) plays an instrumental role in reproductive regulation [59]. The synthesized version, GnRH-a, exhibits a biological effect immensely higher than natural GnRH [60]. Its mode of action involves inhibiting gonadotropin secretion in the pituitary, essentially putting the ovary in a dormant state, which subsequently leads to menopausal-level estrogen concentrations [61].

Contrastingly, ESHRE guidelines are hesitant to recommend GnRH-a as a fertility-enhancing agent. However, in real-world clinical scenarios, its combination with conservative surgery is commonly observed, particularly to address pain and recurrence in endometriosis patients. Several studies present a mixed picture regarding its impact on pregnancy rates, with some indicating no significant benefits [38, 43, 47], while others suggest possible advantages [36, 46].

Two past meta-analyses have explored the impact of GnRH-a regimens on reproductive outcomes, with one emphasizing its benefits in advanced endometriosis stages during IVF treatments [62], However, both analyses had limitations in terms of their scope and included patient profiles [63]. Our meta-analysis draws on data from recent RCTs and randomized clinical trials, with a primary focus on pregnancy rates post-conservative surgery combined with GnRH-a. We aggregated results from 2,485 patients (majority being infertile) across seventeen studies. Our findings lean towards the potential of post-surgery GnRH-a in elevating pregnancy rates and diminishing the average time from surgery to conception, although certain results weren’t statistically significant. The ultimate goal of endometriosis patients is to deliver a live-born baby, not just a clinical pregnancy. In our study, the pregnancy rate was the primary outcome, but the live birth rate was the secondary one, as more than half of the included studies didn’t report the live birth rate, perhaps due to its multifactorial and heterogeneous nature. As mentioned above, endometriosis increases the risk of adverse pregnancy outcomes and obstetric complications. Therefore, more high-quality studies are needed to clarify the situation of live birth rates in patients with endometriosis and to explore the underlying mechanisms.

In addition, the difference in pregnancy outcomes between ART and natural conception after surgery needs to be considered. A systematic review and meta-analysis [64] including nine studies assessed the effect of ovarian endometriomas on ovarian responsiveness and IVF and found that ovarian endometriomas adversely affected extracted oocytes, MII oocytes and total formed embryos, but embryo quality and IVF outcomes were not adversely affected. Nevertheless, ablation of endometriosis with low thermal energy was found to be beneficial for ovarian reserve and postoperative pregnancy rates in a prospective cohort study evaluating the effect of CO2 fiber laser vaporization on subsequent controlled ovarian stimulation among 26 endometriosis patients [65], seeming optimistic about natural conception after surgery. To date, ART for conception is still controversial, due to so many factors need to be taken into account, such as age, the classification and stage of endometriosis, the endometriosis fertility index (EFI) score and other infertility factors [66].

Moderate to severe patients following conservative surgical treatment may require adjuvant drugs to prevent recurrence, such as dienogest and GnRH-a. Patients with fertility needs who have failed to conceive naturally often seek ART. Milder patients often try to conceive naturally without adjuvant medication, and some moderate-to-severe patients have undergone ART directly after surgery. However, none were analyzed as they did not meet the inclusion criteria. These data may have contributed to the variation in conception outcomes.

The observed heterogeneity in outcomes, especially the pregnancy rate, can be traced back to variances in GnRH-a regimens, control groups, conception methods, and study quality. The intragroup heterogeneity of the subgroups analyzed is non-significant (I^2^ = 0), and the same is true for intergroup heterogeneity with *P* values > 0.05. However, the heterogeneity in pregnancy rate was moderate, and we couldn’t identify the possible factors of its origin as follows: in terms of climatological characteristics, it can be from the country/ethnicity, age group, etc.; and statistically, the measurements of the outcome indexes way. Additional high heterogeneity was observed in other outcomes due to the limited number of studies reporting on these aspects. Sensitivity analyses revealed stability in our results, and funnel plots didn’t indicate any noticeable publication bias for the primary outcomes.

In conclusion, while GnRH-a might offer some advantages for women with endometriosis, it’s essential to weigh these benefits against potential risks, especially in long-term GnRH-a regimens [67, 68], it is advisable to consider the side effects and should individualize the prescription. Exploring non-hormonal medication alternatives might also prove valuable in evaluating both efficacy and safety [69].

However, this meta-analysis is not without its limitations. Inherent heterogeneity across studies, different evaluation standards for pregnancy outcomes, and a modest number of included studies with varying quality levels may introduce biases. Furthermore, the inclusion of only English-published studies could also skew the results.

## Conclusions

To summarize, current evidence, albeit limited, hints at the potential of adjuvant GnRH-a post-conservative surgery in improving pregnancy rates for women with endometriosis. Yet, this improvement is marginal and doesn’t extend to other clinical outcomes. The prevailing data is characterized by its scarcity and varying quality, emphasizing the need for more rigorous research. Future investigations should prioritize robust study designs and adequate sample sizes. Ultimately, to solidify the potential benefits of adjuvant GnRH-a following conservative surgery for endometriosis in relation to pregnancy outcomes, we require an influx of well-structured, larger-scale randomized controlled trials.

### Supplementary Information

**Additional file 1.** Search queries.

**Additional file 2.** Specific reasons for exclusions after full-text review.

**Additional file 3.** Quality evaluations.

**Additional file 4.** Basic characteristics of included studies.

**Additional file 5.** Detailed subgroup statistical analyses.

## Data Availability

All data generated or analyzed during this study are included in this published article and its supplementary information files.

## Abbreviations

ART: Assisted reproductive technology
CI: Confidence interval
CM: Chinese herbal medicine
DNG: Dienogest
GnRH-a: Gonadotropin-releasing hormone agonist
GnRH-ant: Gonadotropin-releasing hormone antagonists
ICSI: Intracytoplasmic sperm injection
IUI: Intrauterine Insemination
IVF: In vitro fertilization
NA: Not applicable
RCTs: Randomized controlled trial
RR: Risk ratio
SMD: Standardized mean difference
SO: Stimulates ovulation

## References

[1] Lin YH, Chen YH, Chang HY, Au HK, Tzeng CR, Huang YH (2018). Chronic niche inflammation in endometriosis-associated infertility: current understanding and future therapeutic strategies. Int J Mol Sci.

[2] Taylor HS, Kotlyar AM, Flores VA (2021). Endometriosis is a chronic systemic disease: clinical challenges and novel innovations. Lancet.

[3] Han SJ, Lee JE, Cho YJ, Park MJ, O’Malley BW (2019). Genomic function of estrogen receptor beta in endometriosis. Endocrinology.

[4] Agarwal SK, Chapron C, Giudice LC, Laufer MR, Leyland N, Missmer SA (2019). Clinical diagnosis of endometriosis: a call to action. Am J Obstet Gynecol.

[5] Becker CM, Bokor A, Heikinheimo O, Horne A, Jansen F, Kiesel L (2022). ESHRE guideline: endometriosis. Hum Reprod Open.

[6] Saunders PTK, Horne AW (2021). Endometriosis: etiology, pathobiology, and therapeutic prospects. Cell.

[7] Koninckx PR, Ussia A, Adamyan L, Tahlak M, Keckstein J, Wattiez A (2021). The epidemiology of endometriosis is poorly known as the pathophysiology and diagnosis are unclear. Best Pract Res Clin Obstet Gynaecol.

[8] Horne AW, Missmer SA (2022). Pathophysiology, diagnosis, and management of endometriosis. BMJ.

[9] Garcia-Gomez E, Vazquez-Martinez ER, Reyes-Mayoral C, Cruz-Orozco OP, Camacho-Arroyo I, Cerbon M (2019). Regulation of inflammation pathways and Inflammasome by sex steroid hormones in endometriosis. Front Endocrinol (Lausanne).

[10] Nezhat C, Nezhat F, Nezhat C (2012). Endometriosis: ancient disease, ancient treatments. Fertil Steril.

[11] Zondervan KT, Becker CM, Missmer SA (2020). Endometriosis. N Engl J Med.

[12] Bonavina G, Taylor HS (2022). Endometriosis-associated infertility: from pathophysiology to tailored treatment. Front Endocrinol (Lausanne).

[13] Della Corte L, Di Filippo C, Gabrielli O, Reppuccia S, La Rosa VL, Ragusa R (2020). The burden of endometriosis on women's lifespan: a narrative overview on quality of life and psychosocial wellbeing. Int J Environ Res Public Health.

[14] Eisenberg VH, Decter DH, Chodick G, Shalev V, Weil C (2022). Burden of endometriosis: infertility, comorbidities, and healthcare resource utilization. J Clin Med.

[15] Bhatt DL (2017). Assessment of stable coronary lesions. N Engl J Med.

[16] Bafort C, Beebeejaun Y, Tomassetti C, Bosteels J, Duffy JM (2020). Laparoscopic surgery for endometriosis. Cochrane Database Syst Rev.

[17] Farland LV, Prescott J, Sasamoto N, Tobias DK, Gaskins AJ, Stuart JJ (2019). Endometriosis and risk of adverse pregnancy outcomes. Obstet Gynecol.

[18] Kobayashi H, Kawahara N, Ogawa K, Yoshimoto C (2020). A relationship between endometriosis and Obstetric complications. Reprod Sci.

[19] Salmeri N, Gennarelli G, Vanni VS, Ferrari S, Ruffa A, Rovere-Querini P (2023). Concomitant autoimmunity in endometriosis impairs endometrium-embryo crosstalk at the implantation site: a multicenter case-control study. J Clinical Medicine.

[20] Salmeri N, Farina A, Candiani M, Dolci C, Bonavina G, Poziello C (2022). Endometriosis and impaired placentation: a prospective cohort study comparing uterine arteries doppler pulsatility index in pregnancies of patients with and without moderate-severe disease. Diagnostics.

[21] Doyle JO, Missmer SA, Laufer MR (2009). The effect of combined surgical-medical intervention on the progression of endometriosis in an adolescent and young adult population. J Pediatr Adolesc Gynecol.

[22] Brown J, Pan A, Hart RJ (2010). Gonadotrophin-releasing hormone analogues for pain associated with endometriosis. Cochrane Database Syst Rev.

[23] Arcoverde FVL, Andres MP, Borrelli GM, Barbosa PA, Abrao MS, Kho RM (2019). Surgery for endometriosis improves major domains of quality of life: a systematic review and meta-analysis. J Minim Invasive Gynecol.

[24] Falcone T, Flyckt R (2018). Clinical management of endometriosis. Obstet Gynecol.

[25] Carneiro MM (2023). Deciding on the appropriate pharmacotherapy for the treatment of endometriosis. Expert Opin Pharmacother.

[26] Zakhari A, Delpero E, McKeown S, Tomlinson G, Bougie O, Murji A (2021). Endometriosis recurrence following post-operative hormonal suppression: a systematic review and meta-analysis. Hum Reprod Update.

[27] Bedaiwy MA, Allaire C, Alfaraj S (2017). Long-term medical management of endometriosis with dienogest and with a gonadotropin-releasing hormone agonist and add-back hormone therapy. Fertil Steril.

[28] Tang M, Yang W, Zhang H (2023). Comparison of the efficacy of dienogest and GnRH-a after endometriosis surgery. BMC Womens Health.

[29] Berlanda N, Somigliana E, Vigano P, Vercellini P (2016). Safety of medical treatments for endometriosis. Expert Opin Drug Saf.

[30] Endometriosis Committee, Chinese Obstetricians and Gynecologists Association; Cooperative Group of Endometriosis, Chinese Society of Obstetrics and Gynecology, Chinese Medical Association. [Chinese consensus on the long term management of endometriosis]. Zhonghua Fu Chan Ke Za Zhi. 2018;53(12):836–41. Chinese. 10.3760/cma.j.issn.0529-567x.2018.12.007. 10.3760/cma.j.issn.0529-567x.2018.12.007 30585022

[31] Hodgson RM, Lee HL, Wang R, Mol BW, Johnson N (2020). Interventions for endometriosis-related infertility: a systematic review and network meta-analysis. Fertil Steril.

[32] Alborzi S, Zahiri Sorouri Z, Askari E, Poordast T, Chamanara K (2019). The success of various endometrioma treatments in infertility: a systematic review and meta-analysis of prospective studies. Reprod Med Biol.

[33] DiVasta AD, Feldman HA, Sadler Gallagher J, Stokes NA, Laufer MR, Hornstein MD (2015). Hormonal add-back therapy for females treated with gonadotropin-releasing hormone agonist for endometriosis: a randomized controlled trial. Obstet Gynecol.

[34] Jee BC, Lee JY, Suh CS, Kim SH, Choi YM, Moon SY (2009). Impact of GnRH agonist treatment on recurrence of ovarian endometriomas after conservative laparoscopic surgery. Fertil Steril.

[35] Porpora MG, Pallante D, Ferro A, Crisafi B, Bellati F, Benedetti Panici P (2010). Pain and ovarian endometrioma recurrence after laparoscopic treatment of endometriosis: a long-term prospective study. Fertil Steril.

[36] Ding Y, He Y, TuerXun H, AiLi A, Ji F, Yang X-h (2014). Effects of laparoscopic ovarian endometriosis cystectomy combined with postoperative GnRH-a therapy on ovarian reserve, pregnancy, and outcome recurrence. Clin Exp Obstet Gynecol.

[37] Alborzi S, Hamedi B, Omidvar A, Dehbashi S, Alborzi S, Alborzi M (2011). A comparison of the effect of short-term aromatase inhibitor (letrozole) and GnRH agonist (triptorelin) versus case control on pregnancy rate and symptom and sign recurrence after laparoscopic treatment of endometriosis. Arch Gynecol Obstet.

[38] Busacca M, Somigliana E, Bianchi S, De Marinis S, Calia C, Candiani M, Vignali M (2001). Post-operative GnRH analogue treatment after conservative surgery for symptomatic endometriosis stage III-IV: a randomized controlled trial. Hum Reprod..

[39] Rodriguez-Purata J, Coroleu B, Tur R, Carrasco B, Rodriguez I, Barri PN (2013). Endometriosis and IVF: are agonists really better? Analysis of 1180 cycles with the propensity score matching. Gynecol Endocrinol.

[40] Page MJ, McKenzie JE, Bossuyt PM, Boutron I, Hoffmann TC, Mulrow CD (2021). The PRISMA 2020 statement: an updated guideline for reporting systematic reviews. BMJ.

[41] Ceccaroni M, Clarizia R, Liverani S, Donati A, Ceccarello M, Manzone M (2021). Dienogest vs GnRH agonists as postoperative therapy after laparoscopic eradication of deep infiltrating endometriosis with bowel and parametrial surgery: a randomized controlled trial. Gynecol Endocrinol.

[42] Rodríguez-Tárrega E, Monzo AM, Quiroga R, Polo-Sánchez P, Fernández-Colom P, Monterde-Estrada M (2020). Effect of GnRH agonist before IVF on outcomes in infertile endometriosis patients: a randomized controlled trial. Reproductive biomedicine online.

[43] Loverro G, Carriero C, Rossi AC, Putignano G, Nicolardi V, Selvaggi L (2008). A randomized study comparing triptorelin or expectant management following conservative laparoscopic surgery for symptomatic stage III–IV endometriosis. Eur J Obstet Gynecol Reproductive Biology.

[44] Xue H, Liu M, Hao W, Li Y (2018). Clinical evaluation of laparoscopic surgery combined with triptorelin acetate in patients with endometriosis and infertility. Pak J Med Sci.

[45] Yang Y, Zhu W, Chen S, Zhang G, Chen M, Zhuang Y (2019). Laparoscopic surgery combined with GnRH agonist in endometriosis. J Coll Physicians Surg Pak.

[46] Alkatout I, Mettler L, Beteta C, Hedderich J, Jonat W, Schollmeyer T (2013). Combined surgical and hormone therapy for endometriosis is the most effective treatment: prospective, randomized, controlled trial. J Minim Invasive Gynecol.

[47] Bansal P, Khoiwal K, Malhotra N, Dadhwal V, Sharma A, Deka D (2018). The role of GnRH analogues in improving outcome in women undergoing superovulation and intrauterine insemination after surgical correction of mild endometriosis: a randomized controlled trial. The Eurasian Journal of Medicine.

[48] Surrey ES, Silverberg KM, Surrey MW, Schoolcraft WB (2002). Effect of prolonged gonadotropin-releasing hormone agonist therapy on the outcome of in vitro fertilization-embryo transfer in patients with endometriosis. Fertil Steril.

[49] Rickes D, Nickel I, Kropf S, Kleinstein J (2002). Increased pregnancy rates after ultralong postoperative therapy with gonadotropin-releasing hormone analogs in patients with endometriosis. Fertil Steril.

[50] Vercellini P, Crosignani PG, Fadini R, Radici E, Belloni C, Sismondi P (1999). A gonadotrophin-releasing hormone agonist compared with expectant management after conservative surgery for symptomatic endometriosis. Br J Obstet Gynaecol.

[51] Guo H, Du T, Gao H, Xi Q, Wu L, Lyu Q (2022). The comparison of two different protocols ultra-long versus medroxyprogesterone acetate in women with ovarian endometriosis: a prospective randomized controlled trial. Reproductive Health.

[52] Pabuccu R, Onalan G, Kaya C (2007). GnRH agonist and antagonist protocols for stage I–II endometriosis and endometrioma in in vitro fertilization/intracytoplasmic sperm injection cycles. Fertil Steril.

[53] Zhao RH, Hao ZP, Zhang Y, Lian FM, Sun WW, Liu Y (2013). Controlling the recurrence of pelvic endometriosis after a conservative operation: comparison between Chinese herbal medicine and western medicine. Chin J Integr Med.

[54] Decleer W, Osmanagaoglu K, Verschueren K, Comhaire F, Devroey P (2016). RCT to evaluate the influence of adjuvant medical treatment of peritoneal endometriosis on the outcome of IVF. Hum Reprod.

[55] Muteshi CM, Ohuma EO, Child T, Becker CM (2018). The effect of endometriosis on live birth rate and other reproductive outcomes in ART cycles: a cohort study. Hum Reprod Open.

[56] Soriano D, Adler I, Bouaziz J, Zolti M, Eisenberg VH, Goldenberg M (2016). Fertility outcome of laparoscopic treatment in patients with severe endometriosis and repeated in vitro fertilization failures. Fertil Steril.

[57] Bianchi PHM, Pereira RMA, Zanatta A, Alegretti JR, Motta ELA, Serafini PC (2009). Extensive excision of deep infiltrative endometriosis before in vitro fertilization significantly improves pregnancy rates. J Minim Invasive Gynecol.

[58] Sukur YE, Ozmen B, Yakistiran B, Atabekoglu CS, Berker B, Aytac R (2021). Endometrioma surgery is associated with increased risk of subsequent assisted reproductive technology cycle cancellation; a retrospective cohort study. J Obstet Gynaecol.

[59] Beyer DA, Amari F, Thill M, Schultze-Mosgau A, Al-Hasani S, Diedrich K (2011). Emerging gonadotropin-releasing hormone agonists. Expert Opin Emerg Drugs.

[60] Sharpe-Timms KL, Zimmer RL, Jolliff WJ, Wright JA, Nothnick WB, Curry TE (1998). Gonadotropin-releasing hormone agonist (GnRH-a) therapy alters activity of plasminogen activators, matrix metalloproteinases, and their inhibitors in rat models for adhesion formation and endometriosis: potential GnRH-a-regulated mechanisms reducing adhesion formation. Fertil Steril.

[61] Brown J, Farquhar C (2014). Endometriosis: an overview of cochrane reviews. Cochrane Database Syst Rev.

[62] Cao X, Chang HY, Xu JY, Zheng Y, Xiang YG, Xiao B (2020). The effectiveness of different down-regulating protocols on in vitro fertilization-embryo transfer in endometriosis: a meta-analysis. Reprod Biol Endocrinol.

[63] Georgiou EX, Melo P, Baker PE, Sallam HN, Arici A, Garcia-Velasco JA (2019). Long-term GnRH agonist therapy before in vitro fertilisation (IVF) for improving fertility outcomes in women with endometriosis. Cochrane Database Syst Rev.

[64] Yang C, Geng Y, Li Y, Chen C, Gao Y (2015). Impact of ovarian endometrioma on ovarian responsiveness and IVF: a systematic review and meta-analysis. Reprod Biomed Online.

[65] Ottolina J, Ferrari S, Bartiromo L, Bonavina G, Salmeri N, Schimberni M (2020). Ovarian responsiveness in assisted reproductive technology after CO2 fiber laser vaporization for endometrioma treatment: preliminary data. Minerva Endocrinol.

[66] Huang W, Leng JH, Pei TJ, Li R, Ruan XY, Xu B (2022). [Fertility protection and preservation for patients with endometriosis: a Chinese consensus (2022)]. Zhonghua Fu Chan Ke Za Zhi.

[67] Farmer JE, Prentice A, Breeze A, Ahmad G, Duffy JMN, Watson A (2003). Gonadotrophin-releasing hormone analogues for endometriosis: bone mineral density. Cochrane Database Syst Reviews.

[68] Veth VB, van de Kar MMA, Duffy JMN, van Wely M, Mijatovic V, Maas JWM (2023). Gonadotropin-releasing hormone analogues for endometriosis. Cochrane Database of Systematic Reviews.

[69] Chen FY, Wang X, Tang RY, Guo ZX, Deng YZ, Yu Q (2019). New therapeutic approaches for endometriosis besides hormonal therapy. Chin Med J (Engl).

