# Supplementary material for: Systematic review and meta-analysis on the effect of adjuvant gonadotropin-releasing hormone agonist (GnRH-a) on pregnancy outcomes in women with endometriosis following conservative surgery
Source: BMC Pregnancy Childbirth. 2024 Apr 4;24:237. doi: 10.1186/s12884-024-06430-1 (PMC10993455; doi:10.1186/s12884-024-06430-1)

**Additional file 1: Search queries**

**N=403 (database=399; Other sources：3; uncompleted study：1)**

**PubMed: n=86**

Search: **(((("Endometriosis"[Mesh]) OR (((Endometrioses) OR (Endometrioma)) OR (Endometriomas))) AND (("Surgical Procedures, Operative"[Mesh]) OR ((((((((((((((((Operative Procedures) OR (Operative Procedure)) OR (Procedure, Operative)) OR (Procedures, Operative)) OR (Surgical Procedure, Operative)) OR (Operative Surgical Procedures)) OR (Procedure, Operative Surgical)) OR (Procedures, Operative Surgical)) OR (Surgical Procedures)) OR (Procedure, Surgical)) OR (Procedures, Surgical)) OR (Surgical Procedure)) OR (Operative Surgical Procedure)) OR (Surgery, Ghost)) OR (Ghost Surgery))))) AND (((((((Gonadotropin-releasing hormone agonist) OR (GnRH-a)) OR (Triptorelin)) OR (Goserelin)) OR (Leuprolide)) OR (Ganirelix)) OR (Ganirelix))) AND (("Pregnancy Rate"[Mesh]) OR (((((((((((Rates, Pregnancy) OR (Pregnancy Rates)) OR (Rate, Pregnancy)) OR (Pregnancy Rate, Live-Birth)) OR (Live-Birth Pregnancy Rates)) OR (Pregnancy Rate, Live Birth)) OR (Pregnancy Rates, Live-Birth)) OR (Rate, Live-Birth Pregnancy)) OR (Rates, Live-Birth Pregnancy)) OR (Live-Birth Pregnancy Rate)) OR (Live Birth Pregnancy Rate)))**

**Embase：n=246**

**#13** #9 AND #10 AND #11 AND #12 [246](http://www-embase-com-443.bjmu.ilibs.cn/)

**#12** #7 OR #8 [44,608](http://www-embase-com-443.bjmu.ilibs.cn/)

**#11** #5 OR #6 [21,304](http://www-embase-com-443.bjmu.ilibs.cn/)

**#10** #3 OR #4 [8,367,641](http://www-embase-com-443.bjmu.ilibs.cn/)

**#9** #1 OR #2 [53,458](http://www-embase-com-443.bjmu.ilibs.cn/)

**#8 'rate, pregnancy'**/exp OR **'rate, pregnancy'** OR **'pregnancy rate'**/exp OR **'pregnancy rate'** [44,608](http://www-embase-com-443.bjmu.ilibs.cn/)

**#7 'pregnancy rate'**/exp [39,268](http://www-embase-com-443.bjmu.ilibs.cn/)

**#6 'gnrh agonist'**/exp OR **'gnrh agonist'** OR **'gonadotropin releasing hormone agonist'**/exp OR **'gonadotropin releasing hormone agonist'** OR **'lhrh agonist'**/exp OR **'lhrh agonist'** OR **'luteinising hormone releasing hormone agonist'**/exp OR **'luteinising hormone releasing hormone agonist'** OR **'luteinizing hormone releasing hormone agonist'**/exp OR **'luteinizing hormone releasing hormone agonist'** OR **'gonadorelin agonist'**/exp OR **'gonadorelin agonist'** [21,304](http://www-embase-com-443.bjmu.ilibs.cn/)

**#5 'gonadorelin agonist'**/exp [17,467](http://www-embase-com-443.bjmu.ilibs.cn/)

**#4 'diagnosis, surgical'**/exp OR **'diagnosis, surgical'** OR **'diagnostic techniques, surgical'**/exp OR **'diagnostic techniques, surgical'** OR **'operation'**/exp OR **'operation'** OR **'operation care'**/exp OR **'operation care'** OR **'operative intervention'**/exp OR **'operative intervention'** OR **'operative repair'**/exp OR **'operative repair'** OR **'operative restoration'**/exp OR **'operative restoration'** OR **'operative surgery'**/exp OR **'operative surgery'** OR **'operative surgical procedure'**/exp OR **'operative surgical procedure'** OR **'operative surgical procedures'**/exp OR **'operative surgical procedures'** OR **'operative treatment'**/exp OR **'operative treatment'** OR **'research surgery'**/exp OR **'research surgery'** OR **'resection'**/exp OR **'resection'** OR **'specialties, surgical'**/exp OR **'specialties, surgical'** OR **'surgery, operative'**/exp OR **'surgery, operative'** OR **'surgical care'**/exp OR **'surgical care'** OR **'surgical correction'**/exp OR **'surgical correction'** OR **'surgical diagnosis'**/exp OR **'surgical diagnosis'** OR **'surgical diagnostic techniques'**/exp OR **'surgical diagnostic techniques'** OR **'surgical exposure'**/exp OR **'surgical exposure'** OR **'surgical intervention'**/exp OR **'surgical intervention'** OR **'surgical management'**/exp OR **'surgical management'** OR **'surgical operation'**/exp OR **'surgical operation'** OR **'surgical practice'**/exp OR **'surgical practice'** OR **'surgical procedures, operative'**/exp OR **'surgical procedures, operative'** OR **'surgical repair'**/exp OR **'surgical repair'** OR **'surgical research'**/exp OR **'surgical research'** OR **'surgical restoration'**/exp OR **'surgical restoration'** OR **'surgical service'**/exp OR **'surgical service'** OR **'surgical speciality'**/exp OR **'surgical speciality'** OR **'surgical specialties'**/exp OR **'surgical specialties'** OR **'surgical specialty'**/exp OR **'surgical specialty'** OR **'surgical therapy'**/exp OR **'surgical therapy'** OR **'surgical treatment'**/exp OR **'surgical treatment'** OR **'surgery'**/exp OR **'surgery'** [8,367,641](http://www-embase-com-443.bjmu.ilibs.cn/)

**#3 'surgery'**/exp [6,130,418](http://www-embase-com-443.bjmu.ilibs.cn/)

**#2 'adenomyosis externa'**/exp OR **'adenomyosis externa'** OR **'endometriosis externa'**/exp OR **'endometriosis externa'** OR **'endometriosis'**/exp OR **'endometriosis'** [53,458](http://www-embase-com-443.bjmu.ilibs.cn/)

**#1 'endometriosis'**/exp [48,674](http://www-embase-com-443.bjmu.ilibs.cn/)

**The Cochrane Library：n=22**


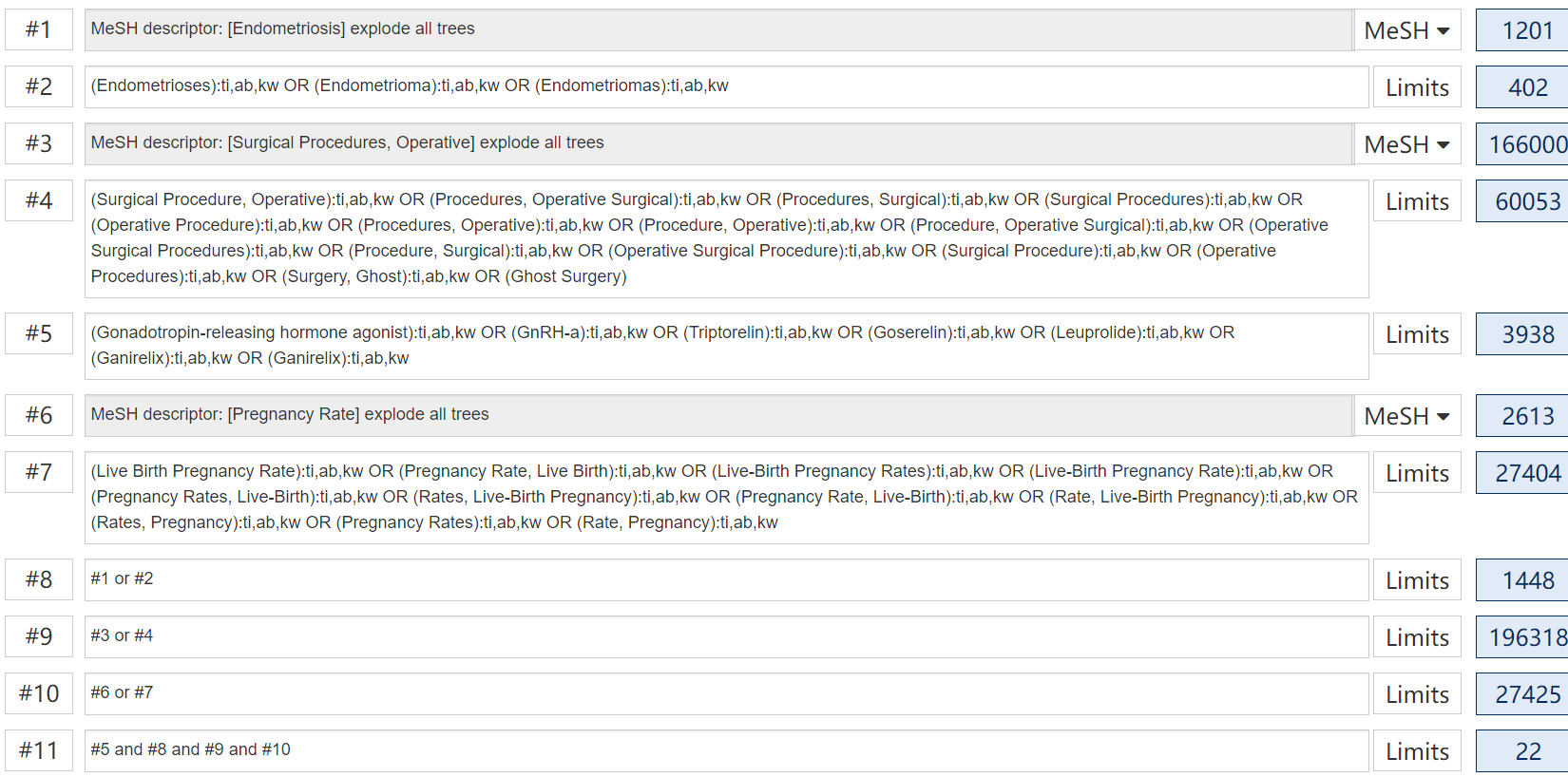


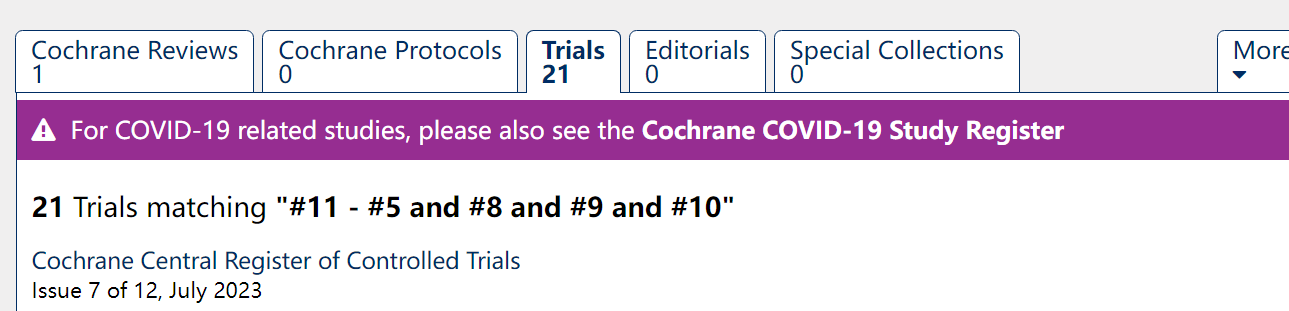


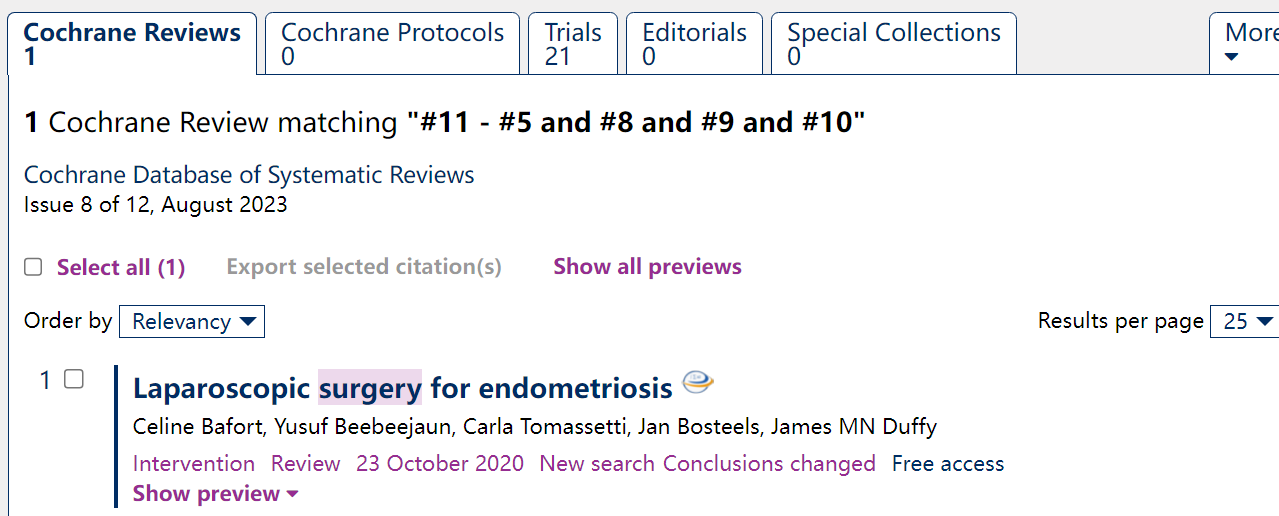


**Web of Science: n=27**

**Search：Endometriosis(Topic) and Surgical Procedures, Operative(Topic) and GnRH-a(Topic) and pregnancy rate(Topic);Refined by Open Access**

**MEDLINE（Ovid）：n=16**

| [# ▲](https://ovidsp.dc2.ovid.com/ovid-a/ovidweb.cgi?&S=JBNBFPKIHHEBFDOAIPLJOHPFIDLDAA00&Sort+Sets=descending) | **Searches** | **Results** | **Type** |
| --- | --- | --- | --- |
| 1 | (Endometriosis or Endometrioses or Endometrioma or Endometriomas).tw. | 29178 | Advanced |
| 2 | (Operative or Surgery).tw. | 1558569 | Advanced |
| 3 | (GnRH-a or Triptorelin or Goserelin or Leuprolide or Ganirelix).tw. | 4970 | Advanced |
| 4 | (pregnancy rate or Live-Birth Pregnancy Rates or Rate, Live-Birth Pregnancy).tw. | 15246 | Advanced |
| 5 | 1 and 2 and 3 and 4 | 16 | Advanced |

**Scopus: n=2**


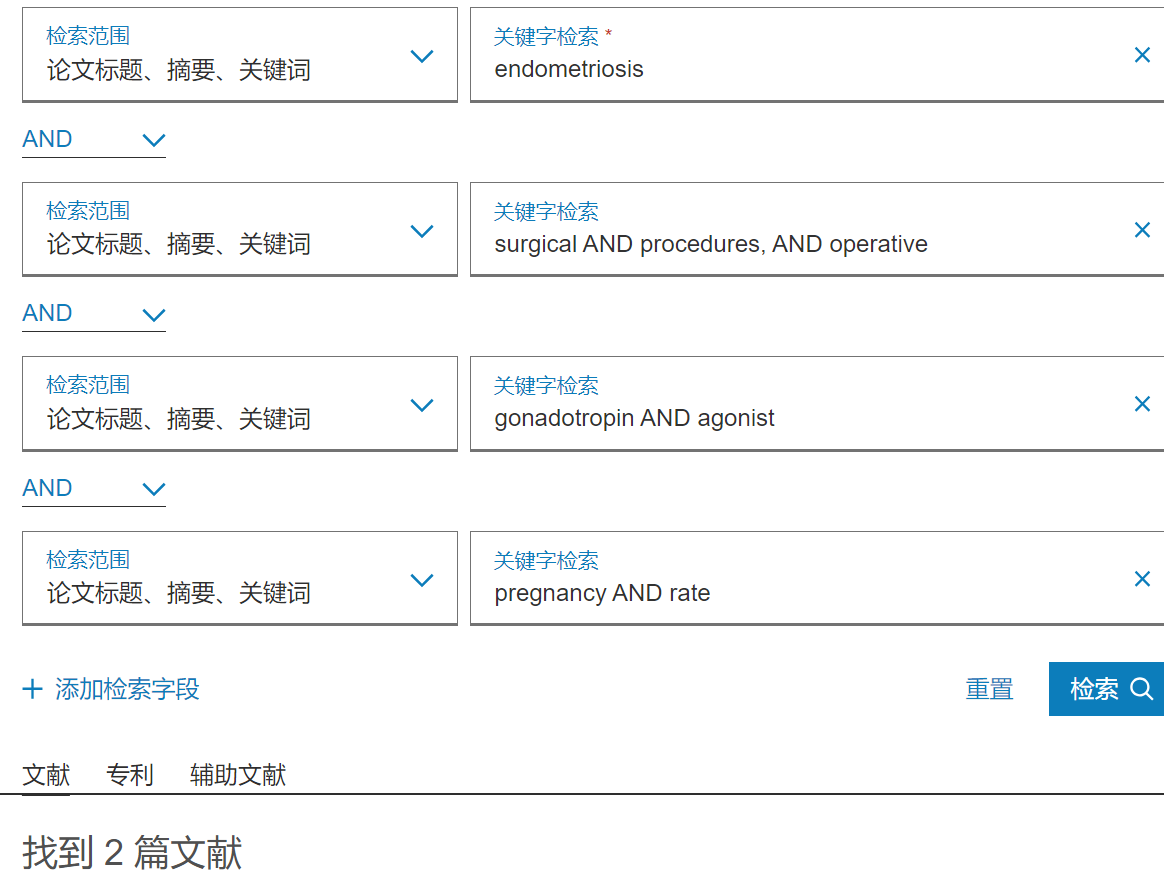

Supplement: Supplementary file 1 — Additional file 1. Search queries. [file 12884_2024_6430_MOESM1_ESM.docx]
