# Supplementary material for: Systematic review and meta-analysis on the effect of adjuvant gonadotropin-releasing hormone agonist (GnRH-a) on pregnancy outcomes in women with endometriosis following conservative surgery
Source: BMC Pregnancy Childbirth. 2024 Apr 4;24:237. doi: 10.1186/s12884-024-06430-1 (PMC10993455; doi:10.1186/s12884-024-06430-1)

**Additional file 5: Detailed subgroup statistical analyses**

Supplementary Figure **1**

Forest plot and Funnel plot of different GnRH-a proposal subgroup analyses on pregnancy rate


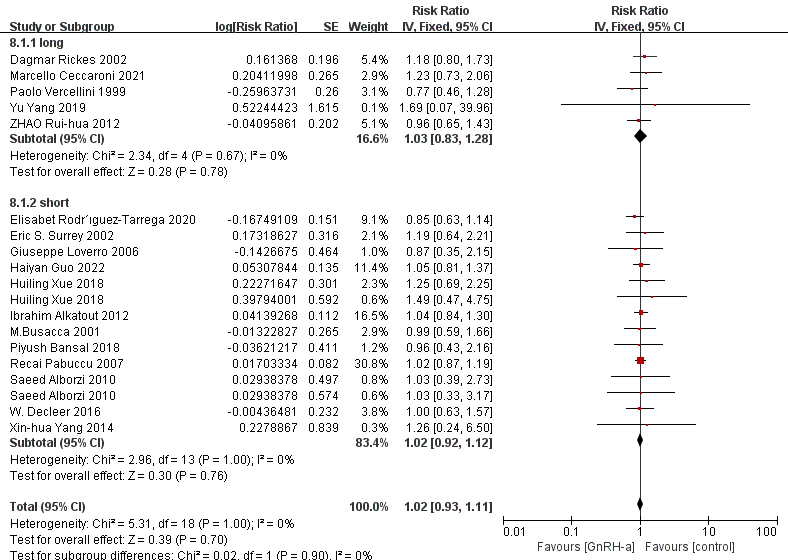


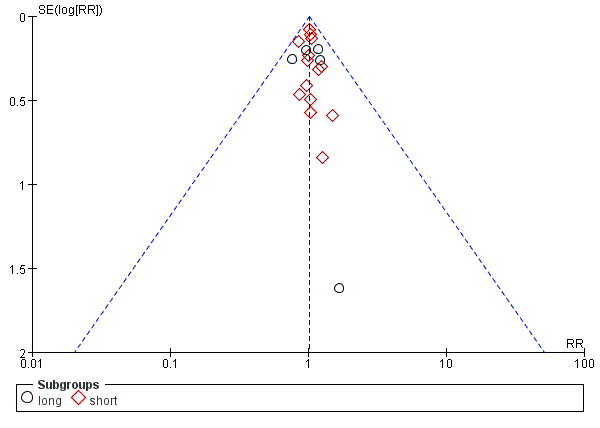


Supplementary Figure **2**

Forest plot and Funnel plot of the different control subgroup analyses on pregnancy rate


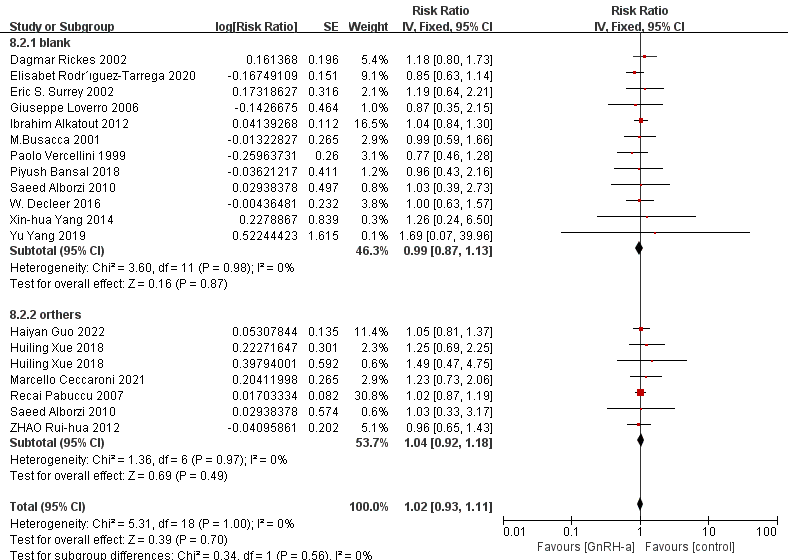


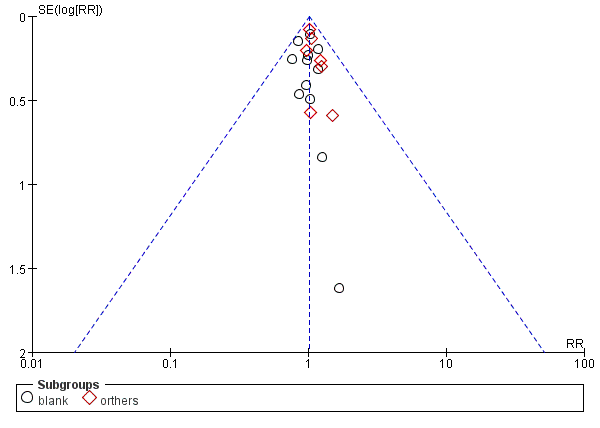


Supplementary Figure **3**

Forest plot and Funnel plot of different conception ways subgroup analyses on pregnancy rate


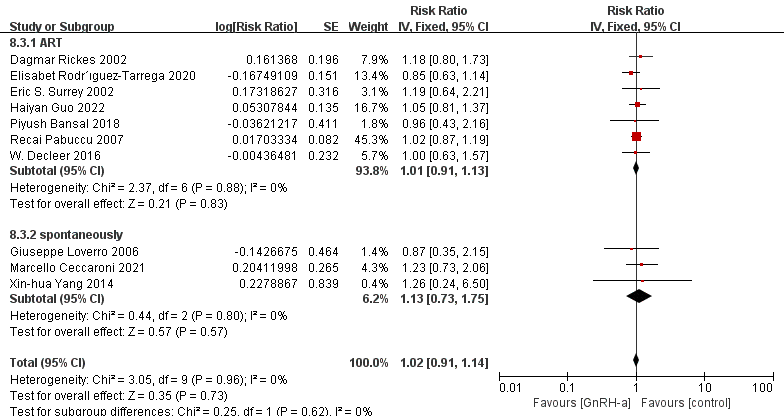


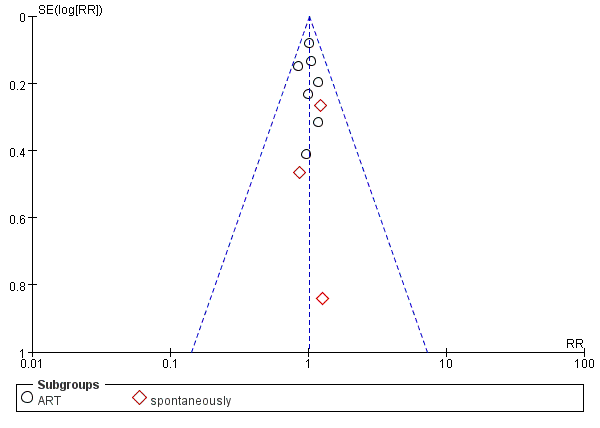


Supplementary Figure **4**

Forest plot and Funnel plot of different study quality subgroup analyses on pregnancy rate


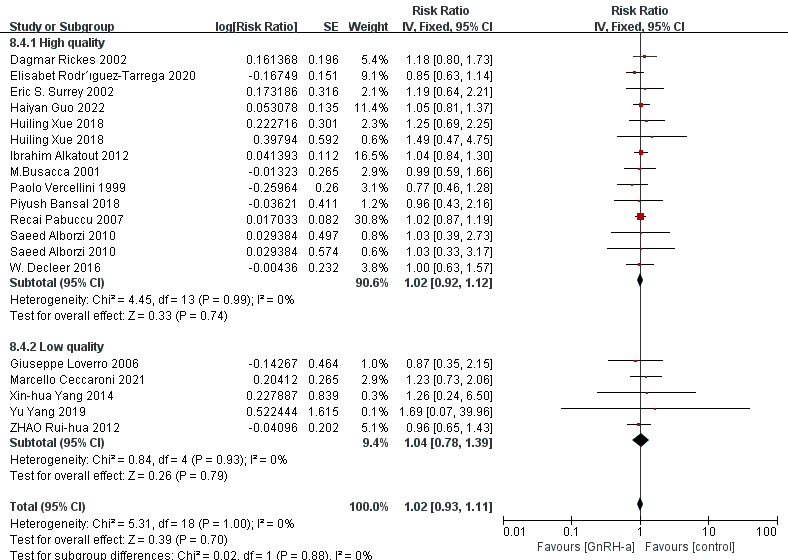


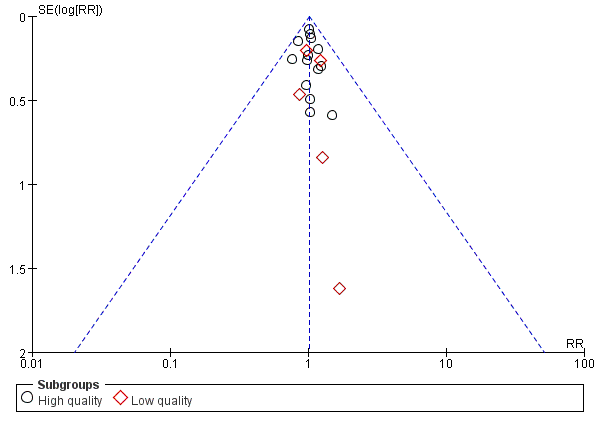


Supplementary Figure **5**

Forest plot and Funnel plot of different GnRH-a proposal subgroup analyses on the live birth rate


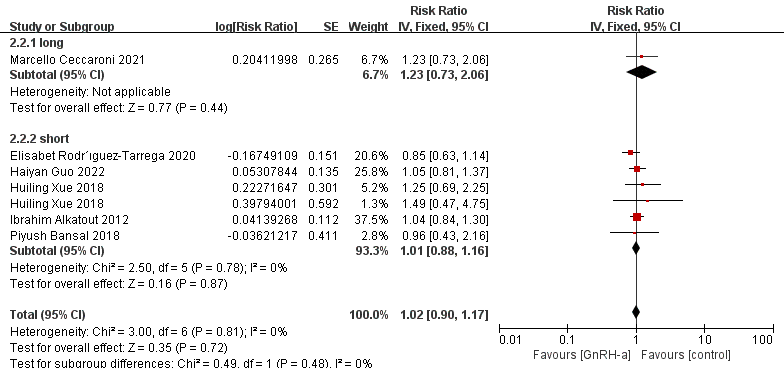


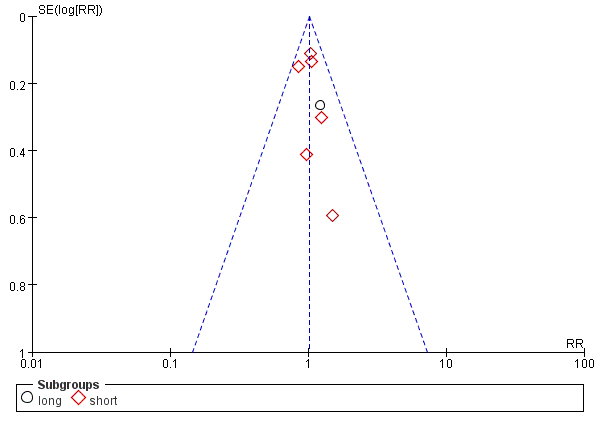


Supplementary Figure **6**

Forest plot and Funnel plot of the different control subgroup analyses on the live birth rate


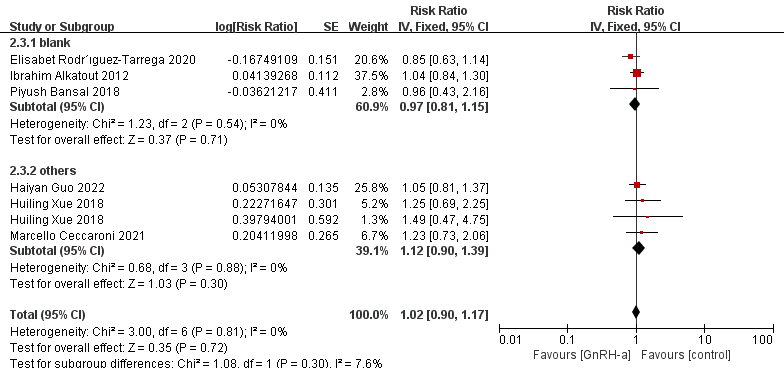


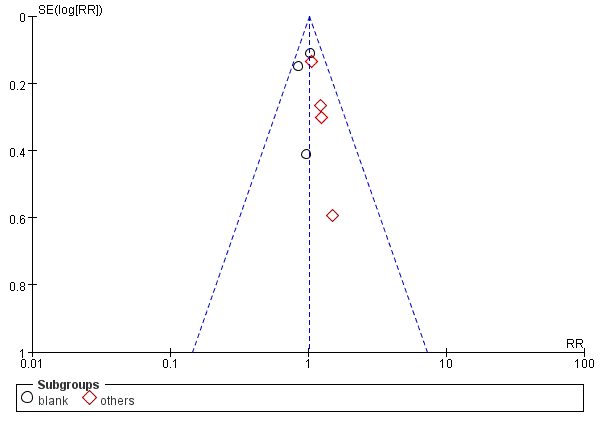


Supplementary Figure **7**

Forest plot and Funnel plot of different conception ways subgroup analyses on the live birth rate


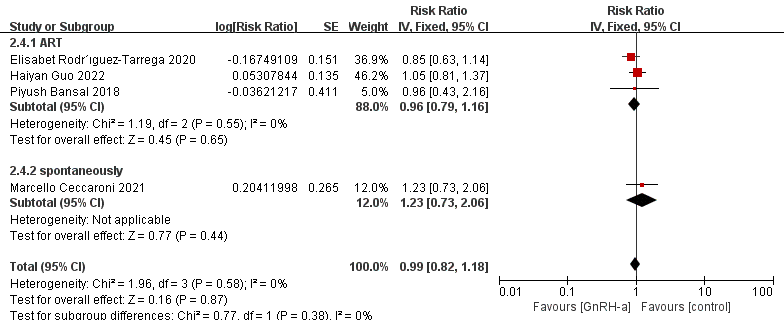


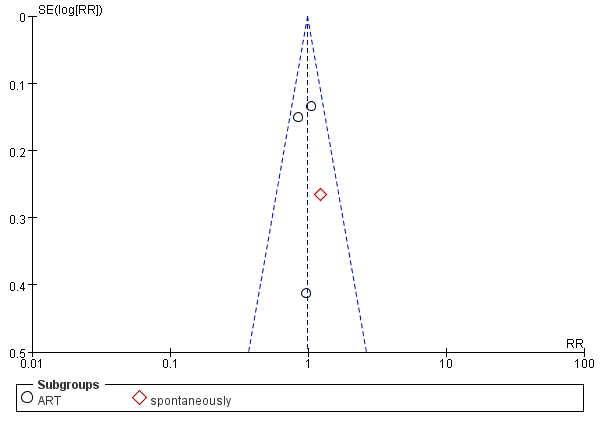


Supplementary Figure **8**

Forest plot and Funnel plot of different study quality subgroup analyses on the live birth rate


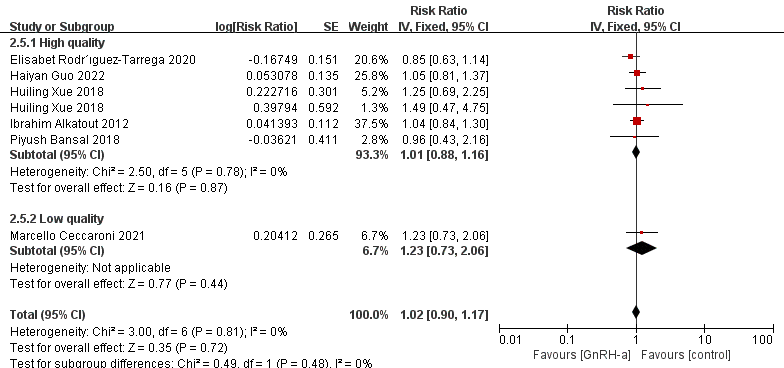


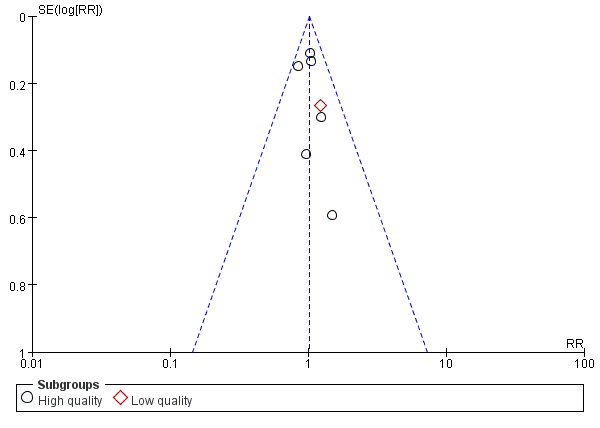

Supplement: Supplementary file 5 — Additional file 5. Detailed subgroup statistical analyses. [file 12884_2024_6430_MOESM5_ESM.docx]
